# FEN1 Blockade for Platinum Chemo-Sensitization and Synthetic Lethality in Epithelial Ovarian Cancers

**DOI:** 10.3390/cancers13081866

**Published:** 2021-04-14

**Authors:** Katia A. Mesquita, Reem Ali, Rachel Doherty, Michael S. Toss, Islam Miligy, Adel Alblihy, Dorjbal Dorjsuren, Anton Simeonov, Ajit Jadhav, David M. Wilson, Ian Hickson, Natalie J. Tatum, Emad A. Rakha, Srinivasan Madhusudan

**Affiliations:** 1 Translational Oncology, Division of Cancer and Stem Cells, School of Medicine, University of Nottingham Biodiscovery Institute, Nottingham NG7 2RD, UK; mesquita.al0602@gmail.com (K.A.M.); reem.ali1@nottingham.ac.uk (R.A.); rejdoherty@aol.com (R.D.); adel.alblihy@nottingham.ac.uk (A.A.); 2 Department of Pathology, Division of Cancer and Stem Cells, School of Medicine, University of Nottingham Biodiscovery Institute, Nottingham NG7 2RD, UK; michael.shawkytoss@nottingham.ac.uk (M.S.T.); islam.abdelaziz@nottingham.ac.uk (I.M.); mrzear1@exmail.nottingham.ac.uk (E.A.R.); 3 Medical Center, King Fahad Security College (KFSC), Riyadh 11461, Saudi Arabia; 4 NIH Chemical Genomics Center, National Center for Advancing Translational Sciences, National Institutes of Health, 9800 Medical Center Drive, Rockville, MD 20850, USA; dorjsurend@mail.nih.gov (D.D.); asimeono@mail.nih.gov (A.S.); ajit.jadhav@gmail.com (A.J.); 5 Biomedical Research Institute, Hasselt University, 3590 Diepenbeek, Belgium; david.wilson@uhasselt.be; 6 Cancer Research UK Newcastle Drug Discovery Unit, Newcastle University Centre for Cancer, Faculty of Medical Sciences, Newcastle University, Newcastle upon Tyne NE2 4HH, UK; Ian.Hickson@newcastle.ac.uk (I.H.); Natalie.tatum@newcastle.ac.uk (N.J.T.); 7 Department of Oncology, Nottingham University Hospitals, City Hospital Campus, Nottingham NG5 1PB, UK

**Keywords:** ovarian cancer, FEN1, cisplatin sensitivity, DNA repair inhibitor, synthetic lethality

## Abstract

**Simple Summary:**

Overall survival outcomes, despite platinum-based chemotherapy, for patients with advanced ovarian cancer remains poor. Increased DNA repair capacity is a key route to platinum resistance in ovarian cancer. In the current study, we show that FEN1, a key player in DNA repair, is overexpressed in ovarian cancer and associated with poor survival. Pre-clinically FEN1 blockade not only increased platinum sensitivity but was also synthetically lethal in BRCA2 and POLβ deficient ovarian cancer cells. Together the data provides evidence that FEN1 is a promising anti-cancer target in ovarian cancer.

**Abstract:**

FEN1 plays critical roles in long patch base excision repair (LP-BER), Okazaki fragment maturation, and rescue of stalled replication forks. In a clinical cohort, FEN1 overexpression is associated with aggressive phenotype and poor progression-free survival after platinum chemotherapy. Pre-clinically, FEN1 is induced upon cisplatin treatment, and nuclear translocation of FEN1 is dependent on physical interaction with importin β. FEN1 depletion, gene inactivation, or inhibition re-sensitizes platinum-resistant ovarian cancer cells to cisplatin. BRCA2 deficient cells exhibited synthetic lethality upon treatment with a FEN1 inhibitor. FEN1 inhibitor-resistant PEO1R cells were generated, and these reactivated BRCA2 and overexpressed the key repair proteins, POLβ and XRCC1. FEN1i treatment was selectively toxic to POLβ deficient but not XRCC1 deficient ovarian cancer cells. High throughput screening of 391,275 compounds identified several FEN1 inhibitor hits that are suitable for further drug development. We conclude that FEN1 is a valid target for ovarian cancer therapy.

## 1. Introduction

Epithelial Ovarian cancer is the third most common gynecologic cancer and is the commonest cause of gynecological cancer death worldwide. Multimodality management strategies including surgery and systemic platinum-based chemotherapy have improved survival outcomes. However, the majority of patients present with advanced-stage disease, will develop platinum resistance, clinical recurrence, and succumb to the disease. Mechanisms of platinum resistance are complex and include upregulation of drug efflux pathways (e.g., modulation of the copper transporter CTR-1 expression), increased levels of proteins that bind and sequester platinum compounds in cells (e.g., GSH), overexpression of the pro-survival protein, or reduction in apoptotic proteins and upregulation of DNA damage signaling and repair pathways. Emerging evidence provides compelling evidence that increased DNA repair capacity in cancer cells is a key route to resistance [1,2]. The cytotoxicity of platinum drugs (carboplatin, cisplatin), commonly used in ovarian cancer therapy, is directly related to the induction of DNA damage in cells. Platinating agents cause intra-strand and inter-strand DNA adducts, which, if unrepaired, can lead to DNA double-strand breaks (DSBs) during replication [1,2]. Platinum drugs can also generate oxygen free radicals [3] that induce oxidative base damage in cells. Whilst nucleotide excision repair (NER) is primarily involved in the repair of intra-strand cross-links [4,5], oxidative base damage is processed through base excision repair (BER) in cells [6,7,8,9].

BER is performed by one of two major sub-pathways: short-patch (SP-BER) or long-patch BER (LP-BER) [6,7,8,9]. Both pathways, in their classic form, are initiated by a damage-specific DNA glycosylase, which removes a substrate base creating an abasic site in duplex DNA. APE1 then cleaves the phosphodiester bond 5′ to the AP site, thereby generating a nick with a 5′-sugar-phosphate (dRP) and 3′-hydroxyl group. DNA polymerase β (POLβ) adds the first nucleotide to the 3′-end of the incised AP site. Most commonly, the reaction continues through SP-BER, where POLβ removes the 5′-sugar-phosphate residue via the process of β-elimination and DNA ligase III-XRCC1 heterodimer (or DNA ligase I) then completes the process by sealing the remaining nick.

In certain situations, such as the processing of oxidized AP sites, a 5′ residue that is resistant to β-elimination mediated by POLβ is generated, therefore requiring strand-displacement DNA synthesis via LP-BER, which may be PCNA (proliferating cell nuclear antigen)-dependent or -independent. In the PCNA-dependent pathway, replication factor C (RF-C) loads PCNA onto DNA, where it functions as a DNA sliding clamp for the polymerases POL δ/ε. Alternatively, LP-BER can proceed via the polymerase activity of POL β under the direction of the Rad9-Rad1-Hus1 sliding clamp complex (9-1-1 complex), which bears structural similarities to PCNA. In both LP-BER processes, the polymerases displace the 5′-sugar-phosphate as part of a generated 2–10 nucleotide flap. The flap is then removed by flap endonuclease 1 (FEN1), and DNA ligase I completes LP-BER by ligating the free DNA ends [6,7,8,9].

FEN1 belongs to XPG/RAD2 endonuclease family, and the *FEN1* gene is located at 11q22. FEN1 possesses flap endonuclease, 5′–3′ exonuclease and gap-endonuclease activities to accomplish its various biological functions [10]. FEN1 is not only critical for processing DNA intermediates generated during LP-BER, but also for Okazaki fragment maturation during replication. In addition, FEN1 is essential for the rescue of stalled replication forks, maintenance of telomere stability, and apoptotic fragmentation of DNA. FEN1 is subjected to post-translational modifications, such as acetylation, phosphorylation, sumoylation, methylation, and ubiquitylation, which appear to regulate nuclease activities as well as protein-protein interactions and subcellular compartmentalization [10].

The role of FEN1 in cancer pathogenesis is complex. *FEN1* homologous knockout in mice is embryonically lethal but *FEN1* heterozygous mice are viable [11]. A double heterozygous mouse model with a mutation in *FEN1* and adenomatous polyposis coli (*APC*) gene showed enhanced cancer development and poor survival [12]. FEN1 E160D mutant mouse model displayed increased mutation frequency and cancer development [13,14]. Polymorphic variants of FEN1 may be associated with increased cancer susceptibility in human studies [15,16]. In established tumors, on the other hand, FEN1 may promote cancer progression and survival [17,18,19,20]. Proliferating cells have been shown to overexpress FEN1 [17]. In pro-myelocytic leukemia cells (HL-60), *FEN1* gene expression was shown to be higher during the mitotic phase compared to the resting phase of the cell cycle and *FEN1* expression markedly decreased upon induction of terminal differentiation in cells [17]. *FEN1* mRNA overexpression has also been demonstrated in lung cancer cell lines [18] and gastric cancer cell lines [19]. In human tumors, frequent overexpression of FEN1 has been reported [21]. In human breast cancers, we have previously shown that FEN1 overexpression at the transcriptional and protein level is linked to aggressive phenotypes and poor survival in patients [22]. In lung cancer, overexpression may have prognostic significance [23] and predict resistance to cisplatin chemotherapy [24]. Similarly, in hepatocellular carcinomas, FEN1 expression may be linked to poor prognosis [25].

Given its various roles in DNA metabolism and the emerging evidence of roles in cancer etiology, we hypothesized a key role for FEN1 in ovarian cancer pathogenesis. In the current study, we provide clinical evidence that FEN1 overexpression is associated with an aggressive phenotype and poor progression-free survival after platinum-based chemotherapy. Pre-clinically, FEN1 depletion or genetic inactivation reversed platinum resistance in ovarian cancer cells. FEN1 small-molecule inhibition increased platinum sensitivity. Importantly, FEN1 inhibition was synthetically lethal in BRCA2 deficient or POLβ deficient ovarian cancer cells. A high throughput screening assay for FEN1 inhibitors identified several novel hits for further development. We conclude that FEN1 is an attractive anti-cancer target in epithelial ovarian cancers, and pharmacological targeting of the nuclease with more advanced small molecule inhibitors is a promising avenue for cancer therapy.

## 2. Results

### 2.1. FEN1 Nuclear Overexpression Is Associated with Clinically Aggressive Epithelial Ovarian Cancers

**Figure 1:**
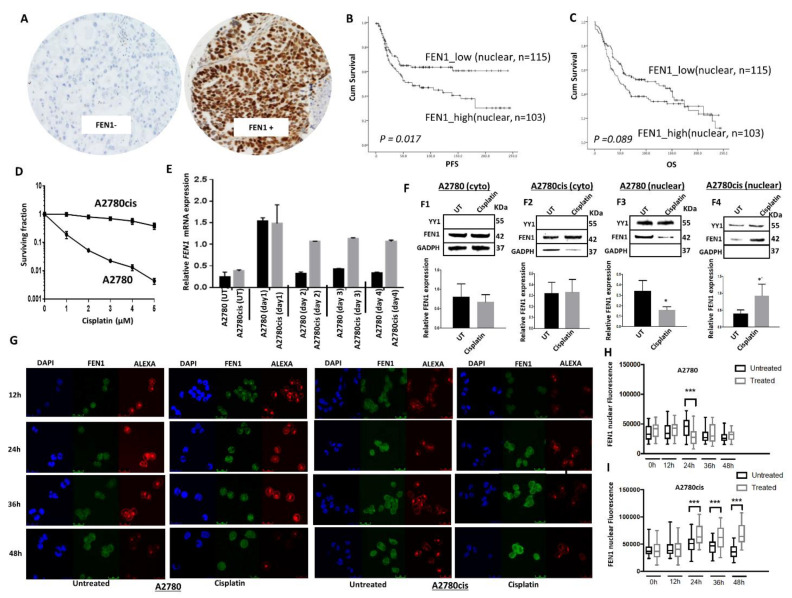
(**A**) Immunohistochemical expression of FEN1 in ovarian cancers. Left side: is ovarian cancer with negative expression (H score 0), right side: is a case with high FEN1 expression (H score 250). (**B**) Kaplan–Meier curve for FEN1 nuclear protein expression and progression-free survival (PFS) in ovarian cancer. (**C**) FEN1 nuclear protein expression and overall survival (OS) in ovarian cancer. (**D**) Clonogenic assay showing cisplatin-sensitive A2780 and cisplatin-resistant A280cis cells (**E**) FEN1 mRNA expression in A2780 and A2780cis cells after cisplatin treatment. (**F**) FEN1 nuclear and cytoplasmic extracts in A2780 and A2780cis treated with 5 µM cisplatin. Lysates were collected 24 h post-treatment and western blot performed for FEN1 expression. (**G**) Con-focal microscopy showing FEN1 nuclear expression following cisplatin treatment at various time points (12 h, 24 h, 36 h, and 48 h). (**H**) Quantification of FEN1 nuclear fluorescence in A2780 cells. (**I**) Quantification of FEN1 nuclear fluorescence in A2780cis cells. * = *p* value < 0.05, *** = *p* value < 0.001.

Patient demographics are summarized in Supplementary Table S1. All patients received platinum-based chemotherapy. A total of 248 tumors were suitable for FEN1 nuclear expression analysis (Figure 1A). 123/248 (49.5%) of tumors showed high FEN1 nuclear expression and 50.5% (125/248) showed low FEN1 nuclear expression (Supplementary Table S1). FEN1 expression within the normal ovarian stroma was either completely negative or showed occasional faint to week staining in the stromal cells. High FEN1 nuclear expression was significantly associated with serous type carcinomas (*p* = 0.018), higher FIGO stage at presentation (*p* = 0.005) and higher tumor grade (*p* = 0.038) (Supplementary Table S2). High FEN1 nuclear expression was also significantly associated with poor progression-free survival (PFS) (*p* = 0.017) (Figure 1B) and was borderline non-significant with overall survival (OS) (*p* = 0.089) (Figure 1C). In addition, we observed high cytoplasmic staining of FEN1 in 116 (47%) of 248 tumors (Supplementary Figure S1A), but cytoplasmic staining did not influence PFS (Supplementary Figure S1B) or OS (Supplementary Figure S1C) of patients. Taken together, the data suggest that FEN1 nuclear expression may predict response to platinum chemotherapy in ovarian cancers, with higher expression associating with reduced treatment effectiveness. We proceeded to pre-clinical studies to explore this hypothesis in detail.

### 2.2. Induction and Altered Sub-Cellular Localization of FEN1 Following Cisplatin Therapy

The A2780 cell line, established from a patient with previously untreated ovarian cancer, is platinum-sensitive, whereas A2780cis is a platinum-resistant ovarian cancer cell line developed by chronic exposure of the parental cisplatin-sensitive A2780 cell line to increasing concentrations of cisplatin. We first confirmed platinum resistance in A2780cis cells compared to A2780 cells (Figure 1D). To explore the role of FEN1 in cisplatin resistance, we monitored FEN1 mRNA expression following cisplatin treatment. As shown in Figure 1E, following 24 h of exposure to cisplatin, there was a transient induction of *FEN1 mRNA* expression on day 1 in A2780 cells that returned to basal levels on day 2. Conversely, in A2780cis cells, following cisplatin exposure, *FEN1 mRNA* expression level increased by day 1 and persisted at least until day 4 (Figure 1E). To determine the expression and subcellular localization of the FEN1 protein following cisplatin treatment, we generated nuclear and cytoplasmic extracts before and after 24 h of genotoxin exposure (Figure 1F). Cytoplasmic levels of FEN1 were not significantly altered in A2780 (Figure 1F1) and A2780cis cells (Figure 1F2) before and after cisplatin treatment. However, the nuclear level of FEN1 significantly decreased in A2780 cells (Figure 1F3), whereas nuclear levels of FEN1 significantly increased in A2780cis cells (Figure 1F4). Using confocal microscopy, we confirmed that in A2780cis cells, increased FEN1 nuclear sub-localization was evident at 24 h and persisted up to 48 h after cisplatin treatment. In A2780 cells, we observed a reduction in FEN1 nuclear fluorescence after cisplatin therapy. Given the key role of FEN1 in DNA repair and replication, the data here provide evidence that FEN1 accumulation in the nucleus of A2780cis cells could contribute to platinum resistance. Conversely, in the A2780 cells, FEN1 translocation to the cytoplasm could lead to platinum sensitivity.

### 2.3. FEN1 Nuclear Localization in Response to Cisplatin in Mediated by Importin β

**Figure 2:**
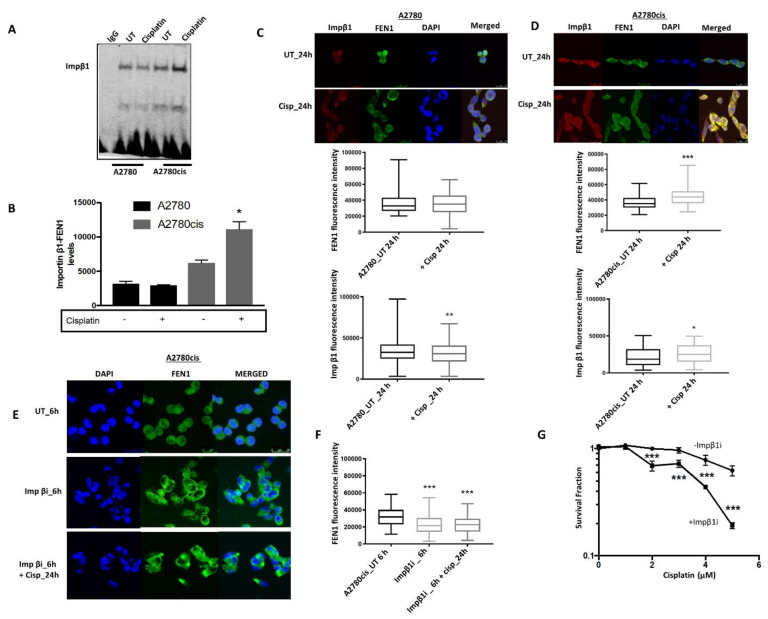
(**A**) Co-IP showing the physical interaction between FEN1 and importin β. The lysate was initially pulled down using FEN1 antibody and later importin β detected using western blots. See methods for details. (**B**) Quantification of FEN1-importinβ protein expression. (**C**) Con-focal microscopy showing FEN1 and importin β expression following cisplatin treatment at 24 h and 48 h in A2780 cells. Quantification of FEN1 nuclear fluorescence performed using ImageJ software. (**D**) Con-focal microscopy showing FEN1 and importin β expression following cisplatin treatment at 24 h and 48 h in A2780cis cells. Quantification of FEN1 nuclear fluorescence performed using ImageJ software. (**E**) Con-focal microscopy showing FEN1 and importin β expression following importin β inhibitor for 6 h followed by cisplatin treatment at 6 h, 24 h, and 48 h. (**F**) Quantification of FEN1 nuclear fluorescence by ImageJ software following importin β inhibitor for 6 h followed by cisplatin treatment at 6 h, 24 h, and 48 h. (**G**) Clonogenic assay showing platinum re-sensitization in A2780cis cells treated with importin β inhibitor and cisplatin compared to cisplatin only. All figures are representative of 3 or more experiments. * = *p* value < 0.05, ** = *p* value < 0.01, *** = *p* value < 0.001

To explore the regulation of FEN1 translocation into the nucleus, we conducted further confocal microscopy studies examining the contributions of specific signaling sequences within the protein. Nuclear import of proteins with nuclear localization signals (NLSs), e.g., FEN1 is mediated by shuttling carriers, the importins [26]. The classic nuclear protein import pathway is orchestrated by the heterodimer importin α/β. Importin β binds to importin α, which in turn binds to a nearby NLS, enabling translocation of proteins into the nucleus [27]. The C-terminal region of FEN1 has an NLS that may facilitate nuclear localization of the protein in response to DNA damage induced by alkylating agents [28]. We evaluated the interaction between FEN1 and importin β in untreated and cisplatin-treated cells by co-immunoprecipitation. As shown in Figure 2A,B, we observed that FEN1 physically interacts with importin β. Moreover, the level of importin β in untreated A2780cis cells was higher compared to A2780 cells. Upon cisplatin treatment, we detected increased levels of importin β in A2780cis cells, while there was no change in importin β levels in A2780cells (Figure 2A,B). To assess this phenomenon at higher resolution, we performed confocal microscopy (Figure 2C,D). As expected, following cisplatin treatment, FEN1 and importin β levels increased in A2780cis cells. Conversely, in A2780 cells, FEN1 and importin β levels decreased following cisplatin treatment (Figure 2C,D). When A2780cis cells were pre-treated with an importin β inhibitor (Importanzole), we observed reduced nuclear localization of FEN1 (Figure 2E,F). Interestingly, pre-treatment of A2780cis cells with the importin β inhibitor re-sensitized these cells to cisplatin therapy (Figure 2G). Together, the data provide evidence that FEN1 protein is increased and translocated to the nucleus after cisplatin treatment, and that FEN1 nuclear localization is mediated through the importin β pathway.

### 2.4. FEN1 Depletion or CRISPR Inactivation Reverses Platinum Resistance in Ovarian Cancer Cells

**Figure 3:**
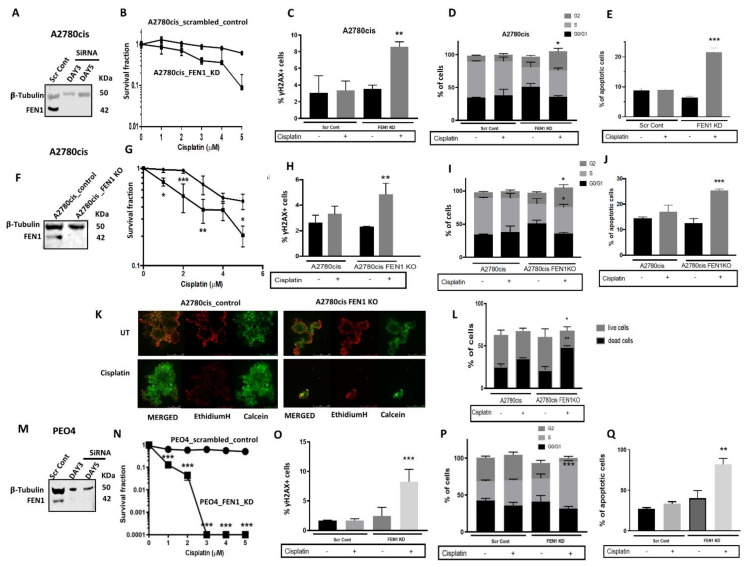
(**A**) FEN1_KD in A2780cis cells. (**B**) Cisplatin sensitivity in A2780cis control and A2780cis_FEN1_KD cells. (**C**) Quantification of γH2AX nuclear fluorescence by ImageJ software. (**D**) Cell cycle analysis by flow cytometry (**E**) Annexin V analysis by flow cytometry. (**F**) FEN1_CRISPR_KO in A2780cis cells. (**G**) Cisplatin sensitivity in A2780cis control and A2780cis_FEN1_KO cells. (**H**) Quantification of γH2AX nuclear fluorescence by ImageJ software. (**I**) Cell cycle analysis by flow cytometry (**J**) Annexin V analysis by flow cytometry. (**K**) Cisplatin sensitivity in A2780cis control and A2780cis_FEN1_KO spheroids. (**L**) Quantification of dead and living cells following cisplatin treatment in A2780cis control and A2780cis_FEN1_KO spheroids. (**M**) FEN1_KD in PEO4 cells. (**N**) Cisplatin sensitivity in PEO4 control and PEO4_FEN1_KD cells. (**O**) Quantification of γH2AX nuclear fluorescence by ImageJ software. (**P**) Cell cycle analysis by flow cytometry (**Q**) annexin V analysis by flow cytometry. All figures are representative of 3 or more experiments. * = *p* value < 0.05, ** = *p* value < 0.01, *** = *p* value < 0.001.

To determine the precise role of FEN1 in cisplatin resistance, we depleted FEN1 using siRNAs in A280cis cells (Figure 3A) and tested platinum sensitivity. FEN1_KD_A2780cis cells (Figure 3B) showed increased platinum sensitivity that was associated with DSB accumulation (Figure 3C), G2/M cell cycle arrest (Figure 3D), and increased apoptosis (Figure 3E). Similarly, FEN1 deficient HeLa SilenciX cells were sensitive to cisplatin compared to control HeLa cells (Supplementary Figure S2A). We also generated A2780cis FEN1-knockout (KO) cells using CRISPR-Cas9 techniques (Figure 3F). FEN1_KO increased platinum sensitivity in A2780cis cells (Figure 3G) that was associated with increased γH2AX nuclear foci accumulation (Figure 3H), G2/M cell cycle arrest (Figure 3I), and accumulation of apoptotic cells (Figure 3J). To recapitulate an in vivo setting, we generated 3D-spheroids of A2780cis control and A2780cis FEN1_KO cells (Figure 3K). Similar to control cells, untreated FEN1_KO cells retain spheroid forming capacity. However, upon cisplatin treatment, FEN1_KO cells exhibited a substantial reduction in spheroid size (Figure 3K), as well as an accumulation of apoptotic cells (Figure 3L).

The PEO4 platinum-resistant ovarian adenocarcinoma cell line was derived from a malignant effusion from the peritoneal ascites of a patient who developed clinical resistance to platinum chemotherapy (Supplementary Figure S2B). To further evaluate the contribution of FEN1 to platinum resistance, we generated transient knockdowns (KD) of FEN1 in PEO4 cells (Figure 3M). In clonogenic assays, FEN1_KD_PEO4 cells (Figure 3N) were significantly more sensitive to platinum therapy than the scramble controls, a phenotype associated with DSB accumulation (Figure 3O), S-phase cell cycle arrest (Figure 3P), and increased apoptosis (Figure 3Q). Taken together, the above pre-clinical data provide strong evidence that FEN1 is an important regulator of platinum sensitivity in ovarian cancer. This conclusion is consistent with our clinical study that found better progression-free survival in patients whose tumors had low FEN1 expression (Figure 1B). In addition, the data suggest that a small molecule blockade of FEN1 could be a promising anti-cancer approach in ovarian cancers.

### 2.5. FEN1 Small Molecule Inhibitor Potentiates Cisplatin Cytotoxicity in Ovarian Cancer Cells

**Figure 4:**
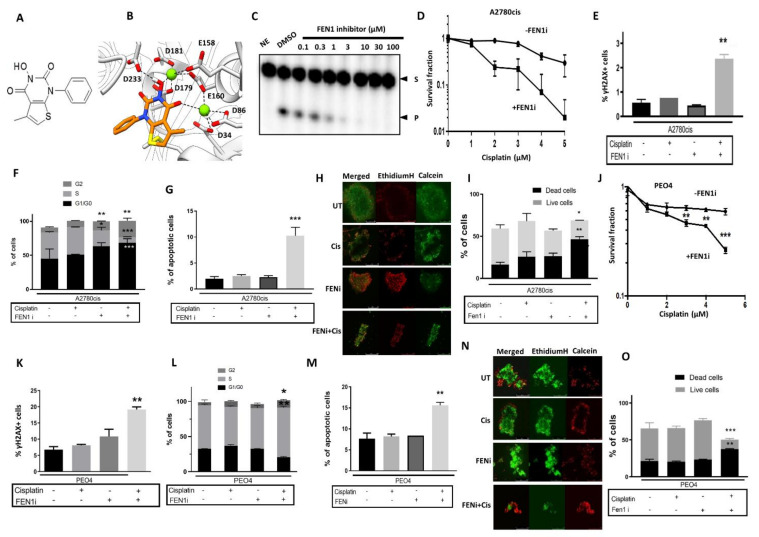
(**A**) Chemical structure of FEN1 inhibitor. (**B**) Docking of FEN1 inhibitor in the FEN1 crystal structure. (**C**) FEN1 cleavage assay. FEN1 inhibitor was added at the indicated concentration. The absence of a lower band indicates FEN1 inhibition. See methods for details. (**D**) Clonogenic assay in cisplatin-treated A2780cis cells with or without FEN1 inhibitor. (**E**) γH2AX analysis by flow cytometry in cisplatin-treated A2780cis cells with or without FEN1 inhibitor. (**F**) Cell cycle progression in cisplatin-treated A2780cis cells with or without FEN1 inhibitor. (**G**) AnnexinV analysis by flow cytometry in cisplatin-treated A2780cis cells with or without FEN1 inhibitor. (**H**) Cisplatin sensitivity A2780cis spheroids with or without FEN1 inhibitor. (**I**) Quantification of dead and living cells in cisplatin-treated A2780cis spheroids with or without FEN1 inhibitor. (**J**) Clonogenic assay in cisplatin-treated PEO4 cells with or without FEN1 inhibitor. (**K**) γH2AX analysis by flow cytometry in cisplatin-treated PEO4 cells with or without FEN1 inhibitor. (**L**) Cell cycle progression in cisplatin-treated PEO4 cells with or without FEN1 inhibitor. (**M**) AnnexinV analysis by flow cytometry in cisplatin-treated PEO4 cells with or without FEN1 inhibitor. (**N**) Cisplatin sensitivity PEO4 spheroids with or without FEN1 inhibitor. (**O**) Quantification of dead and living cells in cisplatin-treated PEO4 spheroids with or without FEN1 inhibitor. All figures are representative of 3 or more experiments. * = *p* value < 0.05, ** = *p* value < 0.01, *** = *p* value < 0.001.

A previously described FEN1 inhibitor, 3-hydroxy-5-methyl-1-phenylthieno [2,3 d]pyrimidine-2,4(1H,3H)-dione) (Figure 4A, also known as PTPD) [29] was evaluated in our pre-clinical ovarian cancer models. PTPD was synthetized as described previously [29] and displays an IC_50_ of 0.022 µM for FEN1 inhibition [29]. A subsequent study identified a related molecule that inhibited FEN1 with an IC_50_ of 0.046 µM, for which the crystal structure of the compound in FEN1 was determined (PDB F5V7) [30]. We have determined that PTPD docking into FEN1 adopts a similar conformation (Figure 4B) and predicts similar mechanistic activity. Following the in-silico modeling, we confirmed FEN1 inhibition using a radio-labeled FEN1 cleavage assay (Figure 4C). The cytotoxicity of FEN1i monotherapy in A2780 and A2780cis cells was evaluated and is shown in Supplementary Figure S2C. At a non-toxic concentration (10 µM), FEN1i significantly enhanced the cytotoxicity of cisplatin in A2780cis cells (Figure 4D). In neutral COMET assays, combination therapy substantially increased DNA breaks compared to cisplatin or FEN1i monotherapy alone (Supplementary Figure S2D). FEN1i plus cisplatin combination in A2780cis cells also induced nuclear γH2AX nuclear foci accumulation (Figure 4E), G2/M cell cycle arrest (Figure 4F) and accumulation of apoptotic cells (Figure 4G). In 3D-spheroids, as expected, the FEN1i/cisplatin combination increased cell death compared to cisplatin or FEN1i monotherapy (Figure 4H,I). In PEO4 cells, FEN1i similarly potentiated cisplatin cytotoxicity (Figure 4J), an outcome associated with increased nuclear γH2AX nuclear foci accumulation (Figure 4K), G2/M cell cycle arrest (Figure 4L) and accumulation of apoptotic cells (Figure 4M). In 3D spheroid models of PEO4 cells, FEN1i/cisplatin combination also increased cell death compared to cisplatin or FEN1i monotherapy (Figure 4N,O).

### 2.6. FEN1 Depletion or Inhibition Is Synthetically Lethal with BRCA2 Deficiency

In BRCA deficient germ-line or platinum-sensitive sporadic epithelial ovarian cancers, PARP inhibitor (Niraparib, Olaparib, and Rucaparib) maintenance therapy substantially improves progression-free survival in patients [31,32,33,34]. However, only about 50% of patients experience a clinical benefit from PARP inhibitor therapy, with intrinsic or acquired resistance limiting the use of PARP inhibitors in ovarian cancers. Thus, the development of alternative synthetic lethality strategies is necessary.

**Figure 5:**
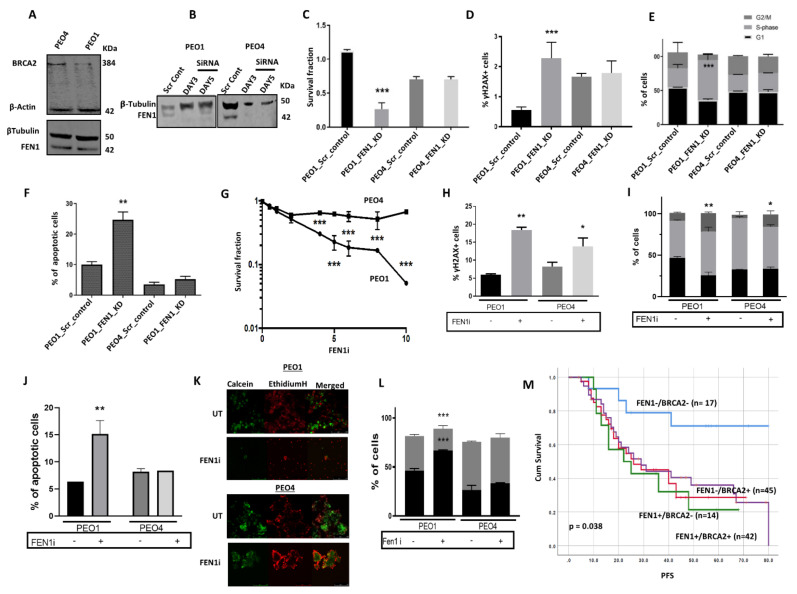
(**A**) Western blot showing BRCA2 and FEN1 expression in PEO1 and PEO4 cells. (**B**) Western blot showing FEN1_KD in PEO1 and PEO4 cells. (**C**) Cell viability was assessed by clonogenic assay in PEO1 or PEO4 control or FEN1_KD cells. (**D**) γH2AX analysis by flow cytometry in PEO1 or PEO4 control or FEN1_KD cells. (**E**) Cell cycle progression in PEO1 or PEO4 control or FEN1_KD cells. (**F**) AnnexinV analysis by flow cytometry PEO1 or PEO4 control or FEN1_KD cells. (**G**) Clonogenic assay showing FEN1 inhibitor sensitivity in PEO1 and PEO4 cells. (**H**) Quantification of γH2AX nuclear fluorescence by ImageJ software in FEN1 inhibitor-treated PEO1 and PEO4 cells. (**I**) Cell cycle analysis by flow cytometry in FEN1 inhibitor-treated PEO1 and PEO4 cells. (**J**) Annexin V analysis by flow cytometry in FEN1 inhibitor-treated PEO1 and PEO4 cells. (**K**) FEN1 inhibitor sensitivity in PEO1 and PEO4 spheroids. (**L**) Quantification of dead and living cells in FEN1 inhibitor-treated PEO1 and PEO4 spheroids. All figures are representative of 3 or more experiments. (**M**) Kaplan-Meier curve for FEN1-BRCA2 co-expression and progression-free survival (PFS) in ovarian cancer. * = *p* value < 0.05, ** = *p* value < 0.01, *** = *p* value < 0.001.

BRCA2, besides its critical role in HR, also protects stalled replication forks through its ability to stabilize RAD51 filaments. FEN1 is not only critical for processing DNA intermediates during LP-BER, but also for Okazaki fragment maturation during replication and the rescue of stalled replication forks. Thus, we speculated that in BRCA2 deficient cells that accumulate replication fork intermediates, blockade of FEN1 nuclease activity would result in the accumulation of toxic DNA intermediates that would promote DSB formation and apoptotic cell death. We, therefore, tested whether FEN1 depletion by siRNA or small molecule inhibition would be synthetically lethal with BRCA2 deficiency. PEO1 is a BRCA2 germ-line deficient ovarian cancer cell line derived from a patient with a poorly differentiated serous adenocarcinoma. The PEO4 cell line was derived from the same patient after the development of resistance to cisplatin chemotherapy. PEO4 is BRCA2 proficient, with the restoration of BRCA2 expression due to secondary gene mutation. Western blot analysis confirmed that PEO1 is BRCA2 deficient and PEO4 is BRCA2 proficient (Figure 5A), and both cell lines were found to have robust FEN1 expression (Figure 5A). Using a siRNA strategy, we successfully depleted FEN1 in PEO1 and PEO4 cells (Figure 5B). Cell viability, as investigated by clonogenic assays, was significantly impaired when FEN1 was depleted in PEO1 cells, but not in PEO4 cells (Figure 5C). The reduced cell viability was associated with increased γH2AX foci accumulation (Figure 5D), S-phase cell cycle arrest (Figure 5E), and induction of apoptosis (Figure 5F) in PEO1 cells. In support of the siRNA findings, we observed that PEO1 cells were also more sensitive to FEN1 inhibition in comparison to PEO4 cells (Figure 5G). The increased sensitivity of PEO1 cells to a FEN1i was associated with increased γH2AX foci accumulation (Figure 5H), S-phase and G2/M-phase cell cycle arrest (Figure 5I), and induction of apoptosis (Figure 5J). Moreover, PEO1 spheroids were also hypersensitive to FEN1 inhibition as compared with PEO4 spheroids (Figure 5K,L). Similarly, in clonogenic assays, BRCA2_KD HeLa silenciX cells were sensitive to FEN1i compared to control HeLa cells (Supplementary Figure S2F). Taken together, the data provide preclinical evidence that FEN1 is a promising synthetic lethality target for BRCA2 deficient ovarian cancers.

In clinical cohorts of ovarian cancers, we observed that tumors with low FEN1/low BRCA2 co-expression were associated with good PFS (*p* = 0.038) compared to tumors with high FEN1/high BRCA2 co-expression (Figure 5M). For overall survival, although tumors with low FEN1/low BRCA2 co-expression had better survival compared to tumors with high FEN1/high BRCA2 co-expression, it was borderline non-significant (*p* = 0.07) (Supplementary Figure S3A).

### 2.7. FEN1i Resistant PEO1R Cells Re-Express BRCA2

**Figure 6:**
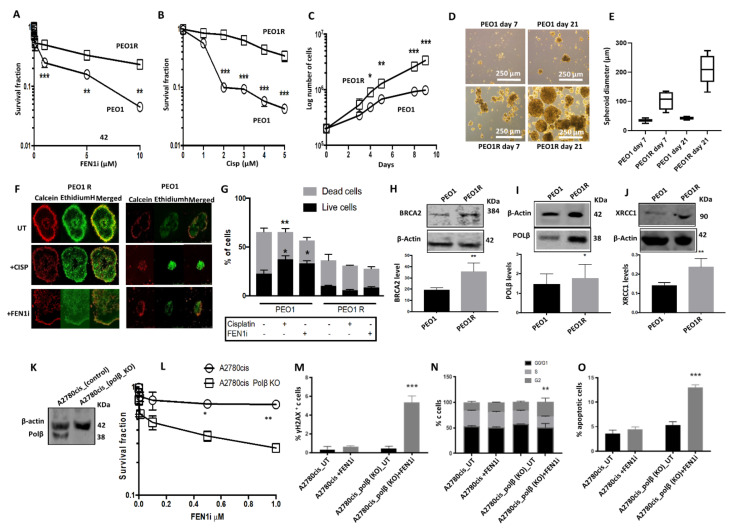
(**A**) Clonogenic assay showing FEN1 inhibitor sensitivity in PEO1 and PEO1R cells. (**B**) Clonogenic assay showing cisplatin sensitivity in PEO1 and PEO1R cells. (**C**) Growth curves showing an increased proliferation rate in PEO1R cells compared to PEO1 cells. (**D**) Representative photomicrographs showing enhanced spheroid formability of PEO1R cells compared to PEO1 cells. (**E**). Quantification of spheroid diameter in PEO1 and PEO1R on day 7 and day 21. A total of 50 spheroids were evaluated for spheroid diameter in PEO1 and PEO1R on day 7 and day 21. (**F**) FEN1 inhibitor sensitivity in PEO1 and PEO1R spheroids. (**G**) Quantification of dead and living cells in FEN1 inhibitor-treated PEO1 and PEO1R spheroids. (**H**) Western blot showing BRCA2 expression in PEO1 and PEO1R cells. (**I**) Western blot showing polβ expression in PEO1 and PEO1R cells. (**J**) Western blot showing XRCC1 expression in PEO1 and PEO1R cells. (**K**) Western blot showing polβ_Knockout (KO) in A2780cis cells using CRISPR methodology [First incubation was with polβ antibody and then imaged. This was followed by incubation with loading control and then imaged for analysis. See Supplementary Materials for details]. (**L**) Clonogenic assay showing FEN1 inhibitor sensitivity in A2780cis control and A2780cis_polβ_KO cells. (**M**) Quantification of γH2AX nuclear fluorescence by ImageJ software in FEN1 inhibitor-treated A2780cis control and A2780cis_polβ_KO cells. (**N**) Cell cycle analysis by flow cytometry in FEN1 inhibitor-treated A2780cis control and A2780cis_polβ_KO cells. (**O**) Annexin V analysis by flow cytometry in FEN1 inhibitor-treated A2780cis control and A2780cis_polβ_KO cells. All figures are representative of 3 or more experiments. * = *p* value < 0.05, ** = *p* value < 0.01, *** = *p* value < 0.001.

PEO1 cells were treated with increasing doses of FEN1i (1 μM–10 μM). At each dose level, PEO1 cells were maintained for three generations following inhibitor exposure. A FEN1i resistant PEO1 cell line (PEO1R) was established over a period of six months. As shown in Figure 6A, the PEO1R cell line was resistant to the FEN1i (Figure 6A) and cisplatin (Figure 6B) treatment compared to the parental PEO1 cells. We also observed that PEO1R cells were significantly more proliferative compared to control cells (Figure 6C). The ability to form spheroids was also evaluated in PEO1R and compared to PEO1 cells. Primary, secondary, and tertiary PEO1R spheroids were significantly larger compared to PEO1 spheroids (Figure 6D,E). Moreover, PEO1R spheroids were resistant to cisplatin and FEN1 inhibition relative to PEO1 spheroids (Figure 6F,G). Studies of PARP inhibitor resistance in BRCA deficient cells have revealed several mechanisms of resistance, including restoration of HR through reactivation of the BRCA function. Interestingly, as shown in Figure 6H, PEO1R cells re-expressed BRCA2, a phenotype that likely contributes to the emergence of resistance to cisplatin and FEN1 inhibition.

### 2.8. FEN1i Is Synthetically Lethal with POLβ Deficient, but Not with a Deficiency in XRCC1, ATM or MRE11

FEN1 is a key player in LP-BER. We explored whether BER upregulation may operate in PEO1R cells compared to PEO1 cells. While there were no significant changes in FEN1 or PARP1 protein levels between the two cell lines (Supplementary Figure S2G), we observed overexpression of POLβ (Figure 6I) and XRCC1 (Figure 6J) in PEO1R cells as compared to its parental counterpart. Since we had previously shown that POLβ or XRCC1 deficiency is a predictor of platinum sensitivity in ovarian cancers [35], we investigated whether a synthetic lethality relationship exists between FEN1 and either POLβ or XRCC1. Towards this end, we first determined the FEN1i sensitivity of POLβ proficient or KO A2780cis cells (Figure 6K). POLβ KO A2780cis cells were not only more sensitive to FEN1 inhibition (Figure 6L), but exhibited increased DSBs (Figure 6M), G2/M cell cycle arrest (Figure 6N), and apoptosis (Figure 6O). Conversely, we did not observe any increased sensitivity to the FEN1i in XRCC1 deficient A2780cis cells (Supplementary Figure S3B) or XRCC1 deficient HeLa cells (Supplementary Figure S3C). In addition, we also did not observe increased sensitivity of ATM-deficient HeLa cells (Supplementary Figure S3D) or MRE11 deficient A2780cis cells (Supplementary Figure S3E) to FEN1 inhibition, relative to their respective controls.

### 2.9. High through-Put Screening (HTS) and Identification of FEN1 Inhibitors

The data presented so far provide evidence that FEN1 targeting is a promising anti-cancer approach. To identify additional small molecule inhibitors of FEN1, we used a previously described fluorescence-based assay [29] (Supplementary Figure S4A) and conducted an HTS on 391,275 compounds arrayed as dilution series within a total of 1407 plates. As seen before, a stable Z′ statistical factor was observed (Supplementary Figure S4B). Moreover, a dilution series of PTPD was included within each screening plate and produced a uniform inhibition pattern throughout the fully automated screen (Supplementary Figure S4C). A representative set of screening hits displaying a range of potencies and chemical structures are shown in Supplementary Figure S4D,E. Full primary screening data has been uploaded to a public database (PubChem ID 588795), and while undeveloped at this time, represents a critical starting point for the development of potent and selective FEN1 inhibitors.

## 3. Discussion

FEN1 has important roles in LP-BER, Okazaki fragment maturation, the rescue of stalled replication forks, the maintenance of telomere stability, and apoptotic fragmentation of DNA [10]. FEN1 overexpression has been commonly observed in cancer lines [17,18,19,20] and human tumors [21]. Adverse prognostic and/or predictive significance of FEN1 has been shown in lung cancer [23,24] and hepatocellular carcinomas [25]. In human breast cancers, we have previously shown that FEN1 overexpression at the transcriptional and protein level is linked to aggressive phenotypes and poor survival in patients [22]. In a small cohort of ovarian cancer, we provided preliminary evidence that FEN1 overexpression may also be associated with poor outcomes in ovarian cancers [22]. In the current study, we validated in a larger cohort of ovarian tumors AND provide evidence that FEN1 overexpression is associated with an aggressive phenotype and predicts platinum resistance in ovarian cancer. The data provides evidence that FEN1 is a promising predictive biomarker in ovarian cancer. In vitro studies reveal that FEN1 is induced upon cisplatin treatment and that nuclear translocation of FEN1 protein is dependent on its interaction with importin β. Moreover, FEN1 depletion or genetic inactivation re-sensitizes platinum resistant ovarian cancer cell lines to cisplatin. Whilst BRCA2 deficiency as a marker of platinum sensitivity has been well described in ovarian cancer cells, the data presented here provides evidence that FEN1 also has important roles in the repair of DNA damage induced by platinum agents. Moreover, the data also suggests that small molecule inhibition of FEN1 could be a potential platinum sensitizer. Accordingly, we tested a prototypical FEN1i and demonstrate that platinum re-sensitization in previously resistant ovarian cancer cells. Notably, we also found that FEN1 inhibition is synthetically lethal in BRCA2 deficient cells, and that reactivation of BRCA2, as well as possibly overexpression of POLβ and XRCC1, may lead to acquired resistance to FEN1i. However, a limitation to the current study is that we have not validated xenograft models. Nevertheless, taken together, our data suggest that FEN1 is a valid target in the treatment of ovarian cancer and that further pharmaceutical development of FEN1 inhibitors is warranted.

Our data would concur with previous studies in LN308 glioma cells and in SGC-7901 gastric cancer cells where FEN1 depletion also increased platinum sensitivity and lead to the accumulation of DSBs [24]. Moreover, in the current study, we have also shown that FEN1 is inducible upon cisplatin therapy and translocate to the nucleus. A previous study demonstrated that FEN1 expression was cell cycle-specific and subcellular localization to the nucleus was evident upon DNA damage. Cook et al. investigated mechanisms of FEN1 nuclear localization and provided evidence that importinα can bind to the C-terminal NLS domain of FEN1 [27] and importin β, in turn, binds to importinα [36]. A novel observation in the current study is that we have shown that FEN1 can also physically interact with importinβ. Moreover, an inhibitor of importinβ blocked FEN1 translocation to the nucleus and re-sensitized A2780cis cells to cisplatin therapy. However, a limitation here is that our data is preliminary. We only included IgG control for Co-IP experiments and show early evidence that FEN1 interacts with importin β and further detailed mechanistic study will be required to confirm our observation of a link between FEN1 and importinβ.

**Figure 7:**
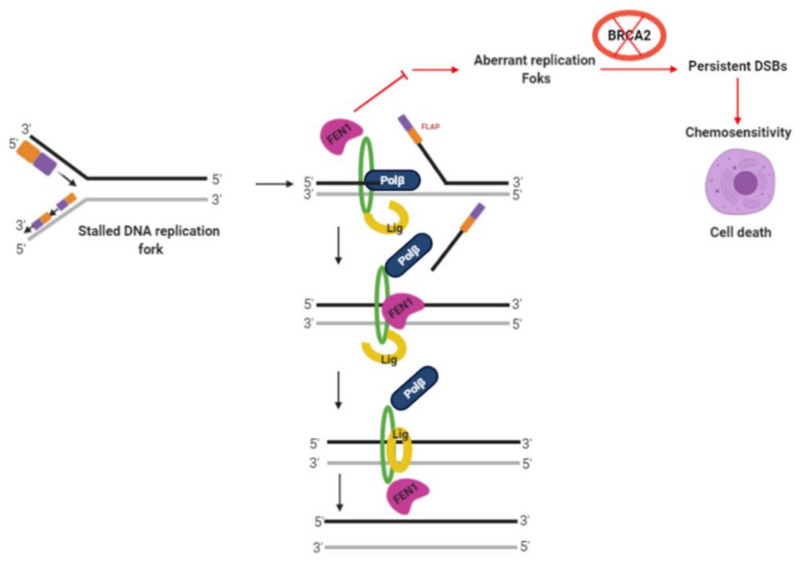
FEN1 and synthetic lethality. BRCA2 deficient cells accumulate replication fork intermediates. FEN1 has an important role during replication. FEN1 blockade in BRCA2 deficient cells will increase replication stress, leading to accumulation of DSBs and cell death. FEN1 also interacts with Polβ during BER. In Polβ deficient ovarian cancer cells, FEN1 blockade can increase SSB accumulation which will lead to DSB accumulation and cell death. DSB = double-strand breaks.

The tumor suppressor BRCA2 is a key player in homologous recombination (HR), a major pathway for the repair of DNA double-strand breaks. In addition, BRCA2 has important roles during replication fork stability [37,38,39,40]. *BRCA2* germline mutations can predispose to ovarian cancer development with a cumulative lifetime risk of about 20–30% [41,42]. PARP inhibitor maintenance therapy improves progression-free survival in BRCA2 germ-line deficient ovarian cancers [43]. However, not all patients respond either due to intrinsic resistance or acquired resistance to PARP inhibitors [44]. The search for alternative DNA repair targets for synthetic lethality is urgently needed. We hypothesized that FEN1 could be a promising alternative synthetic lethality target in BRCA2 deficient ovarian cancers. FEN1 deficiency may not only impair LP-BER but may also affect replication fork stability/progression thereby leading to DSB accumulation and cell death (Figure 7).

We demonstrate herein that FEN1 inhibitor is synthetically lethal in BRCA2 deficient cells. We have concluded synthetic lethality for the following reasons: (1) FEN1 depletion in BRCA2 deficient PEO1 cells reduced viability which was associated with DSB accumulation, S-phase arrest, and increased apoptosis. (2) Second, FEN1 inhibitor increased sensitivity in BRCA2 deficient cells and 3D-spheroids with associated DSB accumulation, cell cycle arrest, and increased apoptosis (Figure 7). Ward et al. provided evidence that FEN1 blockade is selectively toxic in DSB repair impaired cells such as those with MRE11A, ATM, or FANCD2 deficiency [45]. However, in the current study, we did not observe selective toxicity for FEN1 inhibitor in ATM-deficient HeLa cells or MRE11 deficient ovarian cancer cells implying that this phenomenon could be cell line dependent. Nevertheless, the data presented here would concur with a recent study providing evidence that BRCA2 deficient cells require FEN1 endonuclease activity for survival [46]. Mengwasser et al. performed genetic screens in two isogenic (colorectal or ovarian) BRCA2 deficient or proficient cell lines. FEN1 was shown to have a synthetic lethal interaction in BRCA2 deficient cells compared to BRCA2 proficient cells. Increased sensitivity to an N-hydroxyurea-based FEN1 inhibitor was also evident in BRCA2 deficient cells in that study [46]. In the current study, we have confirmed synthetic lethality using a different FEN1 inhibitor (3-hydroxy-5-methyl-1-phenylthieno[2,3 d]pyrimidine-2,4(1H,3H)-dione). We have also confirmed synthetic lethality in 3D- Spheroid models which are more representative of an in vivo system compared to 2D- cell line systems. Importantly, in the current study, we have also elucidated the mechanism of resistance to FEN1 blockade-induced synthetic lethality. Several mechanisms of PARP inhibitor resistance in BRCA deficient cells have been described including restoration of HR through reactivation of BRCA function/expression [44]. To test whether a similar mechanism may evolve during the development of FEN1 inhibitor resistance, we generated a FEN1i resistant PEO1R cell line. PEO1R cells re-expressed BRCA2 which could contribute to cisplatin resistance/FEN1i resistance. The data suggest a mechanism similar to PARP inhibitor resistance described in BRCA deficient cells. Interestingly, PEO1R also overexpressed two key BER proteins; polβ and XRCC1. FEN1 and polβ have been shown to functionally interact with each other during LP-BER. FEN1 can stimulate polβ mediated DNA synthesis during LP-BER (Figure 7). We, therefore, speculate that FEN1 blockade may lead to a compensatory increase in polβ expression which could promote survival. The link between FEN1 and XRCC1 was recently described by Hanzlikova et al. who showed that PARP1 is a sensor of un-ligated Okazaki fragments during DNA replication and PARP1 activation recruits XRCC1 [47]. FEN1 depletion increases un-ligated Okazaki fragments and increases XRCC1 at sites of DNA replication. In XRCC1 deficient RPE-1 cells, FEN1 inhibitor-induced selectively toxicity in that study [47]. We, therefore, investigated if a synthetic lethality relationship also exists between FEN1 and polβ/XRCC1. Although in XRCC1 deficient ovarian cancer cell, FEN1i was not selectively toxic, in polβ_KO ovarian cancer cells FEN1i induced synthetic lethality. The development of inhibitors of POLβ for cancer therapy is an emerging area of investigation in many laboratories including ours. As POLβ_KO ovarian cancer cells were extremely sensitive to FEN1i in our study, we speculate that FEN1i/polβi combinatorial strategies could be a promising approach in ovarian cancer therapeutics. Together, our data not only provide novel insights into the mechanism of resistance to FEN1 inhibitors but also reveal additional synthetic lethality opportunities in epithelial ovarian cancers. In the current study, at the protein level, we also observed that human tumors with low BRCA2/low FEN1 have favorable survival compared to tumors with low BRCA2/high FEN1. In addition to the predictive significance of BRCA2/FEN1 co-expression, the data also suggests that a proportion of ovarian tumors with low BRCA2 may also overexpress FEN1. Whether a similar phenotype may be evident in BRCA2 germ-line deficient human tumors is currently unknown. However, in BRCA2 deficient PEO1 cells we did not observe any overexpression of BRCA2 compared to BRCA2 proficient PEO4 cells.

We conclude that FEN1 is a promising target for platinum chemo-sensitization and synthetic lethality application in ovarian cancers. Pharmaceutical development of FEN1 inhibitors is likely to have a clinical impact on BRCA deficient cancers.

## 4. Methods

### 4.1. Clinical Study

#### FEN1 Protein Level in Ovarian Cancers

Investigation of the expression of FEN1 and BRCA2 in ovarian epithelial cancer was carried out on tissue microarrays of 331 consecutive ovarian epithelial cancer cases treated at NUH between 1997 and 2010. The characteristics of this cohort are summarized in Table S1. None of the tumors were BRCA germ-line deficient. The study was conducted in accordance with the Declaration of Helsinki and ethical approval was obtained from the Nottingham Research Ethics Committee (REC Approval Number 06/Q240/153). Please see Supplementary Materials for Tissue Microarrays (TMAs) and immunohistochemistry (IHC) evaluations.

### 4.2. Pre-Clinical Study

Compounds, reagents, clonogenic assays, cell proliferation assays, confocal microscopy, western blots, co-immunoprecipitation, qRT-PCR, functional assays (neutral COMET assay, FACS, cell cycle progression, apoptosis assay), 3D-spheroid assays, FEN1 cleavage assay, high throughput screening for FEN1 inhibitors and bioinformatics are described in detail in Supplementary Materials.

#### 4.2.1. Cell Lines and Tissue Culture

A2780 (platinum-sensitive) and A2780cis (platinum resistance) human ovarian cancer cell lines were purchased from Sigma Aldrich (Gillingham, UK). PEO1 (BRCA2 deficient) and PEO4 (BRCA2 proficient) were purchased from American Type Culture Collection (ATCC, Manassas, VA, USA). A2780, A2780cis, PEO1, and PEO4 were cultured in RPMI (R8758, Merck, Dorset, UK) with 10% FBS (F4135, Merck, Dorset, UK), 1% Penicillin-Streptomycin (P4333, Merck, Dorset, UK). FEN1-deficient HeLa SilenciX cells and control HeLa cells were purchased from Tebu-Bio and were grown in Dulbecco’s Modified Eagle’s Medium (11965092, Thermo Fisher Scientific) supplemented with 10% FBS, 1% penicillin/streptomycin, and 125 μg/mL hygromycin B. All cell lines were maintained in a humidified incubator at 37 °C in a 5% CO_2_ atmosphere.

#### 4.2.2. Generation of FEN1 Knock-Downs (KD)

For transient KDs, cells were transfected with 20 nM of either FEN1 siRNA oligonucleotide (4390824, Ambion, Thermo Fisher Scientific, Loughborough, UK) or scrambled negative control (4390843, Thermo Fisher, Loughborough, UK). Briefly, 24 h before the transfection, cells were seeded at a density of 8 × 10^3^ cells/cm^2^, approximately 50–60% confluency. The transfection process was made using Lipofectamine 3000 transfection reagent (L3000015, Invitrogen, Loughborough, UK) according to the manufacturer’s instructions. FEN1 KD was checked by western blot. To verify how FEN1 KD influences cell proliferation and cell sensitivity to cisplatin treatment, MTS, Clonogenic, and functional studies were performed after transfection.

#### 4.2.3. CRISPR Editing of FEN1

To generate FEN1 KO cells, the CRISPR/Cas9 methodology was adopted. Cells were transfected with oligonucleotides carrying gRNA silencing FEN1 cloned in a pLV-U6g-EPCG plasmid (Sigma, Gillingham UK). Briefly, cells were seeded at a density of 8 × 10^2^ cells/cm^2^, approximately 50–60% confluency, in 6-well plates overnight. Cells were transfected with 2.5–5 μg of DNA using Lipofectamine 3000 transfection reagent in Opti-MEM medium according to the manufacturer’s instructions. Puromycin (3 μg/mL) was used as a selection marker. Stable FEN1 silencing was checked by western blot and by RT-qPCR.

## Data Availability

Data is avaliable on request.

## Supplementary Materials

The following are available online at https://www.mdpi.com/article/10.3390/cancers13081866/s1. Supplementary Methods; Table S1: Patient demographics and pathological features in ovarian cancer. Table S2. Clinicopathological association between FEN1 and sporadic epithelial ovarian cancers. Figure S1 (A) Immunohistochemical expression of FEN1 (nuclear and cytoplasmic co-expression) in ovarian cancers. (B) Kaplan-Meier curve for FEN1 cytoplasmic protein expression and progression free survival (PFS) in ovarian cancer. (C) Kaplan-Meier curve for FEN1 cytoplasmic protein expression and overall survival (OS) in ovarian cancer. Figure S2 (A) Clonogenic assay showing cisplatin sensitivity in HeLa control and HeLa_FEN1_KD cells. (B) Clonogenic assay showing cisplatin sensitive PEO1 and cisplatin resistant PEO4 cells. (C) Clonogenic assay showing FEN1 inhibitor sensitivity in A2780 and A2780cis cells. (D) Neutral COMET assay in A2780cis cells treated with cisplatin, PTPD or in combination. (E) Clonogenic assay showing FEN1 inhibitor sensitivity in HeLa control and BRCA2 deficient HeLa cells. (F) Western Blot and quantification of PARP1 and FEN1 protein levels in PEO1 and PEO1R cells. Figure S3 A) Kaplan-Meier curve for FEN1/BRCA2 co-expression and overall survival (OS) in ovarian cancer. (B) FEN1 inhibitor cytotoxicity in A2780cis control and A2780cis_XRCC1_KD cells. (C) FEN1 inhibitor cytotoxicity in HeLa control and HeLa _XRCC1_KO cells. (D) FEN1 inhibitor cytotoxicity in HeLa control and HeLa _ATM_KO cells. (E) FEN1 inhibitor cytotoxicity in A2780cis control and A2780cis_MRE11_KD cells. Figure S4 (A) High-throughput screening (HTS) for FEN1 inhibitors. See supplementary methods for details. (B) A stable Z’ statistical factor observed in the FEN1 high-throughput screen. The screen tested 391,275 compounds arrayed as dilution series within a total of 1,407 plates. (C) A dilution series of the FEN1 inhibitor PTPD was included within each screening plate and produced a uniform inhibition pattern throughout the fully-automated screen. (D) A representative set of screening hits displaying a range of potencies: concentration-response curves shown were derived directly from the primary screen, with structures of the indicated hits being available within the publically-deposited screening dataset (PubChem ID 588795). (E) Chemical structures of certain promising hits are shown here.
