# Supplementary material for: FEN1 Blockade for Platinum Chemo-Sensitization and Synthetic Lethality in Epithelial Ovarian Cancers
Source: Cancers (Basel). 2021 Apr 14;13(8):1866. doi: 10.3390/cancers13081866 (PMC8070745; doi:10.3390/cancers13081866)

Clinical Study

FEN1 protein level in ovarian cancers: Investigation of the expression of FEN1 and BRCA2 in ovarian epithelial cancer was carried out on tissue microarrays of 331 consecutive ovarian epithelial cancer cases treated at NUH between 1997 and 2010. The characteristics of this cohort are summarized in Table S1. None of the tumors were BRCA germ-line deficient. The study was conducted in accordance with the Declaration of Helsinki and ethical approval was obtained from the Nottingham Research Ethics Committee (REC Approval Number 06/Q240/153).

Tissue Microarrays (TMAs) and immunohistochemistry (IHC) evaluation: Tumors were arrayed in tissue microarrays (TMAs) constructed with 2 replicate 0.6mm cores from the tumors. Immunohistochemical staining was performed using the Thermo Fisher Scientific Shandon Sequenza chamber system (REF: 72110017), in combination with the Novolink Max Polymer Detection System (RE7280-K: 1250 tests), and the Leica Bond Primary Antibody Diluent (AR9352), each used according to the manufacturer's instructions (Leica Microsystems). The tissue slides were deparaffinised with xylene and then rehydrated through five decreasing concentrations of alcohol (100%, 90%, 70%, 50% and 30%) for two minutes each. Pre-treatment antigen retrieval was performed on the TMA sections using sodium citrate buffer (pH 6.0) and heated for 20 minutes at 95°C in a microwave (Whirlpool JT359 Jet Chef 1000W). A set of slides were incubated with the primary anti-FEN1 mouse monoclonal antibody (NB100-150), at a dilution of 1:100, overnight at 4°C. A set of TMA sections were stained with anti-BRCA2 (Abcam, UK) (1:100), incubated for 1h at room temperature. Negative (by omission of the primary antibody and IgG-matched serum) and positive controls were included in each run.

Whole field inspection of the core was scored and intensities of nuclear staining were grouped as follows: 0 = no staining, 1 = weak staining, 2 = moderate staining, 3 = strong staining. The percentage of each category was estimated (0-100%). Histochemical score (H-score) (range 0-300) was calculated by multiplying intensity of staining and percentage staining. A median H score of > 10 and > 0 was utilised as the cut-off for high FEN1 nuclear and cytoplasmic expression respectively. While, H-score < 120 were considered low/ negative for nuclear BRCA2 expression. Not all cores within the TMA were suitable for IHC analysis as some cores were missing or lacked tumour.

Statistical analysis: Association with clinical and pathological parameters using categorised data was examined using Chi-squared test. All tests were 2-tailed. Survival rates were determined using Kaplan–Meier method and compared by the log-rank test. All analyses were conducted using Statistical Package for the Social Sciences (SPSS, version 22, Chicago, IL, USA) software for windows. P values of less than 0.05 was identified as statistically significant.

<u>Pre-clinical Study</u>

Cell lines and tissue culture: A2780 (platinum sensitive) and A2780cis (platinum resistance) human ovarian cancer cell lines were purchased from Sigma Aldrich (Gillingham, UK). PEO1 (BRCA2 deficient) and PEO4 (BRCA2 proficient) were purchased from American Type Culture Collection (ATCC, Manassas, USA). A2780, A2780cis, PEO1 and PEO4 were cultured in RPMI (R8758, Merck, UK) with 10% FBS (F4135, Merck, UK), 1% Penicillin-Streptomycin (P4333, Merck, UK). FEN1-deficient HeLa SilenciX cells and control HeLa cells were purchased from Tebu-Bio and were grown in Dulbecco's Modified Eagle's Medium (11965092, Thermo Fisher Scientific) supplemented with 10% FBS, 1%

penicillin/streptomycin, and 125 μg/mL hygromycin B. All cell lines were maintained in a humidified incubator at 37°C in a 5% CO_2 atmosphere.

Generation of FEN1 knock downs (KD): For transient KDs**, c**ells were transfected with 20 nM of either FEN1 siRNA oligonucleotide (4390824, Ambion, Thermo Fisher Scientific) or scrambled negative control (4390843, Thermo Fisher). Briefly, 24h before the transfection, cells were seeded at a density of 8×10^3 cells/cm^2, approximately 50- 60 % confluency. Transfection process was made using Lipofectamine 3000 transfection reagent (L3000015, Invitrogen) according to the manufacturer's instructions. FEN1 KD was checked by western blot. To verify how FEN1 KD influences cell proliferation and cells sensitivity to cisplatin treatment, MTS, Clonogenic and functional studies were performed after transfection.

CRISPR editing of FEN1: In order to generate FEN1 KO cells, CRISPR/Cas9 methodology was adopted. Cells were transfected with oligonucleotides carrying gRNA silencing FEN1 cloned in pLV-U6g-EPCG plasmid (Sigma, UK). Briefly cells were seeded at a density of 8×10^2 cells/cm^2, approximately 50-60% confluency, in 6-well plates overnight. Cells were transfected with 2.5-5 μg of DNA using Lipofectamine 3000 transfection reagent in Opti-MEM medium according to the manufacturer's instructions. Puromycin (3 μg/ml) was used as a selection marker. Stable FEN1 silencing was checked by western blot and by RT-qPCR.

MTS cell proliferation assays: In the proliferation assay, 3125 cells/cm^2 were seeded in 96-well plate and left at 37°C in a 5% CO_2 atmosphere. Following day, cisplatin (provided by Nottingham University City Hospital) and/or FEN1i were added up to 48h. MTS assay was performed as per manufacturer's recommendation.. The absorbance was recorded at 490 nm in a 96-well plate reader.

Clonogenic assay: In the clonogenic assay (colony formation assays), 32 cells/cm^2 were seeded in 6-well plates and left at 37°C in a 5% CO_2 atmosphere. Cisplatin (5µM) and/or FEN1i (10µM) and/or Importazole (5 µM, Selleckem, S8446) were added at the indicated concentrations and the plates were left at 37°C in a 5% CO_2 atmosphere for 14 days. Later, the plates were washed with PBS, fixed and stained with a crystal violet, acetic acid and methanol solution and colonies were counted.

Nuclear and cytoplasmic extract: Cells were harvest by trypsinization, washed with PBS and centrifuged at 1000×g for 5min. Nuclear and cytoplasmic lysates were extracted using the NE-PER Nuclear and Cytoplasmic Extraction Reagents from Thermo Fisher Scientific. Protein level were then quantified prior to use in western blots.

BCA protein quantification: Protein quantification was done using the Pierce BCA kit assay by Thermo Fisher Scientific. The standard curve was done with BSA (working range 25 to 2000 µg/mL) following manufacturer's instructions. Then, samples were added to a 96-well micro plate and 200 µL of working reagent (50 parts of BCA Reagent A with 1 part of BCA Reagent B) was added either to samples or standard curve. The plate was incubated at 37°C during 30 min in the dark and the absorbance was measured by FLUOstar OPTIMA, UK/ Infinite® F50 (UK) microplate reader at 590 nm. Standards and unknown samples were performed in duplicate.

Western Blot Analysis: Cells were harvested and lysed in RIPA buffer (R0278, Sigma) with the addition of protease cocktail inhibitor (P8348, Sigma), phosphatase inhibitor cocktail 2 (P5726, Sigma) and phosphatase inhibitor cocktail 3 (P0044, Sigma) and stored at -20°C. Protein was quantified using BCA Protein Assay kit (23225, Thermo Fisher). The following antibodies were used: FEN1 (1:1000, NB100-150), ß-III Tubulin (1:1000, ab6046), YY1 (1:1000, ab109228), GADPH (1:1000, AB9485), NPT97 (1:2000, ab2811) and polβ

(1:200, ab26343). Infrared dye-labelled secondary antibodies (Li-Cor) [IRDye 800CW Donkey Anti-Rabbit IgG and IRDye 680CW Donkey Anti-Mouse IgG] were incubated at a dilution of 1:10000 for 1 h. Membranes were scanned with a LiCor Odyssey machine (700 and 800 nm) to determine protein expression. ß-Tubulin or ß-actin was used as a loading controls for the whole cell lysates. GADPH and YYI were used as loading controls to cytoplasmic and nuclear fractions, respectively. First incubation was with FEN1 antibody and then imaged. This was followed by incubation with loading control and then imaged for analysis. Bar charts show means of optical density (O.D.) ± S.D and all data is representative of at least three independent experiments.

qRT-PCR analysis of FEN1 gene expression: RNA was extracted using the RNeasy Mini Kit (74104, Qiagen) and quantified using a Nanodrop 2000c (Thermo Fisher Scientific). cDNA synthesis was performed using the RT^2 First Strand Kit (330404, Qiagen). Real time PCR was carried out on an Applied Biosystems 75000 FAST cycler.

Neutral COMET assay: 1.0×10^5 cells per well were seeded in 6 well tissue culture plates and incubated at 37°C and 5% CO_2 overnight. Cells were harvested by trypsinization at 6 and 24 hours' post drug administration and neutral COMET assay was performed as described previously (26). Fifty comets were scored per sample using Comet Assay III/IV image analysis software (Perceptive Instruments, Bury St Edmonds, UK). Graphical representation and statistical analysis of mean tail moment was performed in GraphPad Prism 6 (GraphPad, La Jolla, USA). All experiments were done in duplicate three times.

γH2AX FACS: For monitoring DNA double strand break accumulation by γH2AX FACS, 1.05×10^4 Cells/cm^2 were seeded in 6- well plates and left overnight at 37°C in a 5% CO_2 atmosphere. Cisplatin (5µM) or FEN1i (10µM) was later added to cells and incubated for 24h and 48h at 37°C in a 5% CO_2 atmosphere. Cells were collected by trypsinization, washed

with ice cold PBS and fixed in 70% ethanol for 1h at -20°C. After removal of the fixative solution by centrifugation, cells were stained with 2 μg/ml of phospho-Histone (γH2AX) Ser139 (16202A, Millipore, UK) to detect DSBs.

Cell cycle progression: Cells were treated with 20 μg/ml RNase A (12091021, Invitrogen) and then 10 μg/ml Propidium Iodide (P4170, Sigma Aldrich) was added to determine the cell cycle distribution. The samples were analysed on a Beckman-Coulter FC500 flow cytometer using a 488nm laser for excitation and emission data for PI collected using a 620nm bandpass filter (FL3) and a 525nm bandpass filter (FL1) for FITC-anti-phospho-Histone H2A.X.

Apoptosis detection: Annexin V detection kit (556547, BD Biosciences) was used to quantify apoptotic cells. Briefly, cells were trypsinized, washed with PBS and the cellular pellet was re-suspended in Anexin Binding Buffer (1×). Following, FITC Anexin V and Propidium Iodide were added to the cells. After incubation, Anexin binding Buffer (1×) was added to each tube. All the samples were analysed on a Beckman-Coulter FC500 flow cytometer. The percentage of apoptotic cells was determined (late apoptotic = FITC-Annexin V positive, PI positive early apoptotic = FITC-Annexin V positive, PI negative; live cells = FITC-Annexin V negative, PI negative) and compared for untreated and treated samples. Graphical representation and statistical analysis was performed in GraphPad Prism 7 (GraphPad, La Jolla, USA).

3D Spheroid assays: A2780_control and A2780 FEN1_ KO as well as A2780cis_control and A2780cis FEN1_ KO cells were seeded at 4500 cells/cm^2 density in ultra-low attachment 6-well plates (174932, Thermo Fisher Scientific) and cultured in 3D Tumorsphere Medium (C28070, PromoCell, UK) at 37°C in a 5% CO_2 atmosphere. Cells were keep in culture for 7-10 days, toping up with fresh medium every two days until spheroids structures were formed.

For PEO1 and PEO4 cells, the cells where keep in culture up to four weeks (31 days) until spheroids structures were formed.

To quantify cell viability, LIVE/DEAD Viability/Cytotoxicity Kit (L3224, Thermo Fisher Scientific) was used. Briefly, the spheroids were harvest by trypsinization, washed with PBS and centrifuge at 1000×g for 5 minutes. The light-protected cellular pellet in PBS was loaded with 0.1µM of Calcein-AM and 1 µM of Ethidium homodimer-1 for 20 minutes at room temperature. The samples were analysed on a Beckman-Coulter FC500 flow cytometer using a 495nm laser for excitation and a 515nm laser for emission data for Calcein AM and a 495nm laser for excitation and emission at 635nm for Ethidium Homodimer-1. In addition, Image J software was used to calculate spheroid diameter. Mean of three diagonal diameters was taken as diameter for each spheroid. At least 10 spheroids were measured.

Confocal microscopy: Cells were seeded on the cover slips overnight then treated with Cisplatin (5µM), or FEN1i (10µM) or Importin β inhibitor (Importanzole, Selleckem, S8446) (5 µM) for the indicated time points. The cells were fixed with 4% paraformaldehyde (8187085000, Sigma) for 30 mins and washed with PBS. The cells were incubated with 5 µg/ml Alexa Fluor 594 nm for 15 minutes (W11262, Thermo Fisher Scientific) and finally washed. Then cells were permeabilized with 0.1% Triton (85111, Thermo Fisher Scientific) for 30 mins and blocked with 3% BSA (A7906, Sigma) for 1 hr. Cells were incubated with primary antibody FEN1 (1:200, NB100-150, Novus Biologicals) or with Importin ß (1:100, ab2811) overnight at 4°C. In the next day fixed cells were washed with PBS and incubated on dark with 10µg/ml of anti-mouse secondary antibody (A24501, Thermo Fisher Scientific) or with 4µg/ml of anti-mouse secondary antibody (A24501, Thermo Fisher Scientific) for 1 hour at room temperature and washed again with PBS. Slides were prepared in duplicates and mounted in Vectashield Antifade mounting medium with DAPI (H1200, Vector

Laboratories). Imaging was carried out using Leica SP2 confocal laser scanning microscope (CLSM) and analysed with ImageJ software (NIH, USA). For analysis a minimum of 100 cells per slide were counted.

On 3D structures, the spheroids were fixed with 4% paraformaldehyde for 30 minutes. After fixation, spheroids were washed carefuly and a solution in PBS of 2μM of Calcein AM and 1 μM of Ethidium Homodimer-1 was added to the 3D structures. The spheroids were keepped in the dark at room temperature for 1h. Imaging was carried out using Leica SP2 confocal laser scanning microscope (CLSM) and analysed with ImageJ software (NIH, USA).

Immunoprecipitation: For the immunoprecipitation assay PureProteome Protein A/G Mix magnetic beads (LSKMAGA10, Millipore) were used as per and manufacturer's instructions. Briefly, sample and FEN1 capture antibody (antibody-antigen complex) were incubated overnight at 2-8°C with continuous mixing. The buffer from the suspended beads was removed. The beads were washed with washing buffer (PBS, 0,1% Tween 20, pH 7.4). After the removal of the washing buffer from the beads, the antibody-antigen complex was added do dry beads and incubated for 2h at room temperature. After the immune complex was captured, the beads were washed again. Finally, native and denaturing elution were tested to confirm the appropriate elution buffer. The beads were re-engaged to the magnet stand and the supernatant was collected. The samples were checked by western blot.

FEN1 cleavage assay: Recombinant human FEN1 protein was purified from bacteria as described previously (27). To measure FEN1 activity, 3 nM FEN1 protein was mixed with 1 pmol 5'-[^{32}P]-labeled 34(10)FLAP complex (GGTAGGTAAACGTACGGATCCCCGGGTAC) annealed with 15P (CTGCAGCTGATGCGC**)** and 34G (GTACCCGGGGATCCGTACGGCGCATCAGCTGCAG**)** oligonucleotides in 10 μl

reactions containing 50 mM Tris pH 8.0, 10 mM $MgCl_2$, 1 mM DTT and 0.01% Tween-20. Reactions were incubated at 37ºC for 10 min. To inactivate the enzyme, an equal volume of stop buffer (90% Formamide, 20 mM EDTA) was added and the mixture was heated at 95ºC for 10 min. Following separation on a 15% polyacrylamide-urea denaturing gel, substrate and product bands were visualized by a Typhoon Trio+ Variable Model Imager (Amersham Bioscience/GE Healthcare, Piscataway, NJ), and the signals were quantified using ImageQuant software (Molecular Dynamics, GE Healthcare).

Development of HTS assay to isolate novel FEN1 inhibitors: To facilitate the search for novel FEN1 inhibitors, we have developed a fluorogenic donor/quencher reporter pair to monitor generation of reaction product in real time (28) (**Supplementary Figure S4A**). Recombinant human FEN1 protein was purified from bacteria as described previously (27). All oligodeoxynucleotides were purchased from Biosearch Technologies, Inc., (Novato, CA, USA). The double-stranded DNA substrate containing a double flap region used in the fluorogenic assay was prepared from three oligodeoxynucleotides: quencher (5´-CAC GTT GAC TAC CGC TCA ATC CTG ACG AAC ACATC-BHQ-2), flap (5´-TAMRA-GA TGT CAA GCAGTC CTA ACT TTG AGG CAG AGT CCG C) and template (5´-GC GGA CTC TGC CTC AAG ACGGTA GTC AAC GTG-3´). Annealing of the oligodeoxynucleotides was completed in 50mM Tris pH 8.0, 100mM KCl, 5mM MgCl2 by first incubating the mixture at 95°C for 5 min, followed by gradual cooling to room temperature. If the DNA is cleaved at the flap by FEN1, 6-TAMRA fluorophore-containing oligonucleotide will dissociate from its complement by thermal melting. As a result, the quenching effect of the BHQ-2 quencher (which absorbs 6-TAMRA fluorescence when in close proximity) is lost, and FEN1 activity is measured indirectly as an increase in fluorescence signal (Figure 3). Kinetic fluorescence data were collected on ViewLux high-throughput CCD imager (Perkin Elmer, Waltham, MA, USA) equipped with standard optics (excitation filter 525nm and

emission filter 598 nm). FEN1 inhibition by a small molecular inhibitor would be indicated by absence of fluorescence. A large-scale high throughput screening of 391,275 compounds was conducted.

Docking of FEN1i in crystal structure of FEN1: The crystal structure of FEN1 (PDB 5FV7) was used, cleaned and missing loops rebuilt to give the core FEN1 protein covering aa1-336. The 3D conformer of PTPD was created from SMILES by OpenBabel and the docking conducted in MOE using an induce fit model. Three solutions were provided of which the best agreement is shown in Figure 4A with the precedent binding mode.

Statistical analysis: Data are means values ± SD from at least three separate experiments. Graphical representation and statistical analysis was performed in GraphPad Prism 7 (GraphPad, La Jolla, USA). In statistical analysis to compare A2780 and A2780cis the Student's t test was used. For comparisons of more than two groups an ANOVA one-way (variances analyses) with no matched pairs but with multiple comparisons test was used. A two-way ANOVA was used to analyses two variables (e.g. time and drug effect), with same considerations previously explained. The multiple comparisons were made using Bonferroni post-hoc test or (Holm-) Sidak´s test using selected comparisons.

Supplementary Table S1: Patient demographics and pathological features in ovarian cancer.

Characteristics		Number	Percentages
Pathology			
	Serous cystadenocarcinoma	178	53.9%
	Endometrioid	44	13.2%
	Clear cell carcinoma	24	7.2%
	Mucinous cystadenocarcinoma	46	13.8%
	Others	15	4.5%
	Mixed	17	5.1%
Grade			
	1	46	13.8%
	2	60	18%
	3	171	51.5%
Residual tumour			
	None/Microscopic	205	61.7%
	<1cm	34	10.2%
	>1-2 cm	15	4.5%
	>2cm	40	12%
FIGO Stage			
	I	123	37%
	II	49	14.7%
	III	128	38.5%
	IV	11	3.3%
Chemotherapy			
	Carboplatin monotherapy	81	24.3%
	Carboplatin + Paclitaxel	86	25.9%
Platinum sensitivity			
	Sensitive	250	75.3%
	Resistant	26	7.8%
	Unknown	55	16.5%
Relapse status			
	Progression-free	169	50.9%
	Progressed/relapsed	118	35.5%
	Unknown	44	13.2%

Supplementary Table S2. Clinicopathological association between FEN1 and sporadic epithelial ovarian cancers.

**FEN1 expression**						
	N- (%)	N+ (%)	**P- value**	C- (%)	C+ (%)	**P- value**
Pathological Type						
Serous cystadenocarcinoma	59 (41)	85 (59)	***0.018***	63 (43.8)	81 (56.2)	***0.013***
Mucinous cystadenocarcinoma	21 (63.6)	12 (36.4)		23 (69.7)	10 (30.3)	
Endometrioid	23 (67.6)	11 (32.4)		21 (61.8)	13 (38.2)	
Clear cell carcinoma	11 (64.7)	6 (35.3)		13 (76.5)	4 (23.5)	
Mixed	8 (61.5)	5 (38.5)		7 (53.8)	6 (46.2)	
Others	3 (42.9)	4 (57.1)		5 (71.4)	2 (28.6)	
FIGO Stage						
I	61 (62.9)	36 (37.1)	***0.005***	55 (56.7)	42 (43.3)	0.625
II	17 (44.7)	21 (55.3)		17 (44.7)	21 (55.3)	
III	38 (38)	62 (62)		51 (51)	49 (49)	
IV	4 (57.0)	4 (42.9)		4 (57.1)	3 (42.9)	
Tumour Grade						
G1	25 (67.6)	12 (32.4)	***0.038***	21 (56.8)	16 (43.2)	0.765
G2	22 (47.8)	24 (52.2)		24 (52.2)	22 (47.8)	
G3	62 (47)	70 (53)		66 (50.0)	66 (50.0)	

Residual Tumour						
None/Microscopic	90 (57.3)	67 (42.7)	*0.1*	92 (57.1)	69 (42.9)	0.628
<1cm	11 (35.5)	20 (64.5)		15 (48.4)	16 (51.6)	
1-2 cm	5 (45.5)	6 (55.5)		5 (41.7)	7 (58.3)	
>2cm	13 (43.3)	17 (56.7)		17 (56.7)	13 (43.3)	

SUPPLEMENTARY FIGURE LEGENDS

Supplementary Figure S1: **(A)** Immunohistochemical expression of FEN1 (nuclear and cytoplasmic co-expression) in ovarian cancers. **(B)** Kaplan-Meier curve for FEN1 cytoplasmic protein expression and progression free survival (PFS) in ovarian cancer. **(C)** Kaplan-Meier curve for FEN1 cytoplasmic protein expression and overall survival (OS) in ovarian cancer.

Supplementary Figure S2: **(A)** Clonogenic assay showing cisplatin sensitivity in HeLa control and HeLa_FEN1_KD cells. **(B)** Clonogenic assay showing cisplatin sensitive PEO1 and cisplatin resistant PEO4 cells. **(C)** Clonogenic assay showing FEN1 inhibitor sensitivity in A2780 and A2780cis cells. **(D)** Neutral COMET assay in A2780cis cells treated with cisplatin, PTPD or in combination. **(E)** Clonogenic assay showing FEN1 inhibitor sensitivity in HeLa control and BRCA2 deficient HeLa cells. (**F)** Western Blot and quantification of PARP1 and FEN1 protein levels in PEO1 and PEO1R cells.

Supplementary Figure S3: (A) Kaplan-Meier curve for FEN1/BRCA2 co-expression and overall survival (OS) in ovarian cancer. **(B)** FEN1 inhibitor cytotoxicity in A2780cis control and A2780cis_XRCC1_KD cells. **(C)** FEN1 inhibitor cytotoxicity in HeLa control and HeLa _XRCC1_KO cells. (**D**) FEN1 inhibitor cytotoxicity in HeLa control and HeLa _ATM_KO cells. (**E**) FEN1 inhibitor cytotoxicity in A2780cis control and A2780cis_MRE11_KD cells.

Supplementary Figure S4: **(A)** High-throughput screening (HTS) for FEN1 inhibitors. See supplementary methods for details. **(B)** A stable Z' statistical factor observed in the FEN1 high-throughput screen. The screen tested 391,275 compounds arrayed as dilution series within a total of 1,407 plates. **(C)** A dilution series of the FEN1 inhibitor PTPD was included within each screening plate and produced a uniform inhibition pattern throughout the fully-automated screen. **(D)** A representative set of screening hits displaying a range of potencies: concentration-response curves shown were derived directly from the primary screen, with

structures of the indicated hits being available within the publically-deposited screening dataset (PubChem ID 588795). **(E)** Chemical structures of certain promising hits are shown here.

Supplementary Figure S1

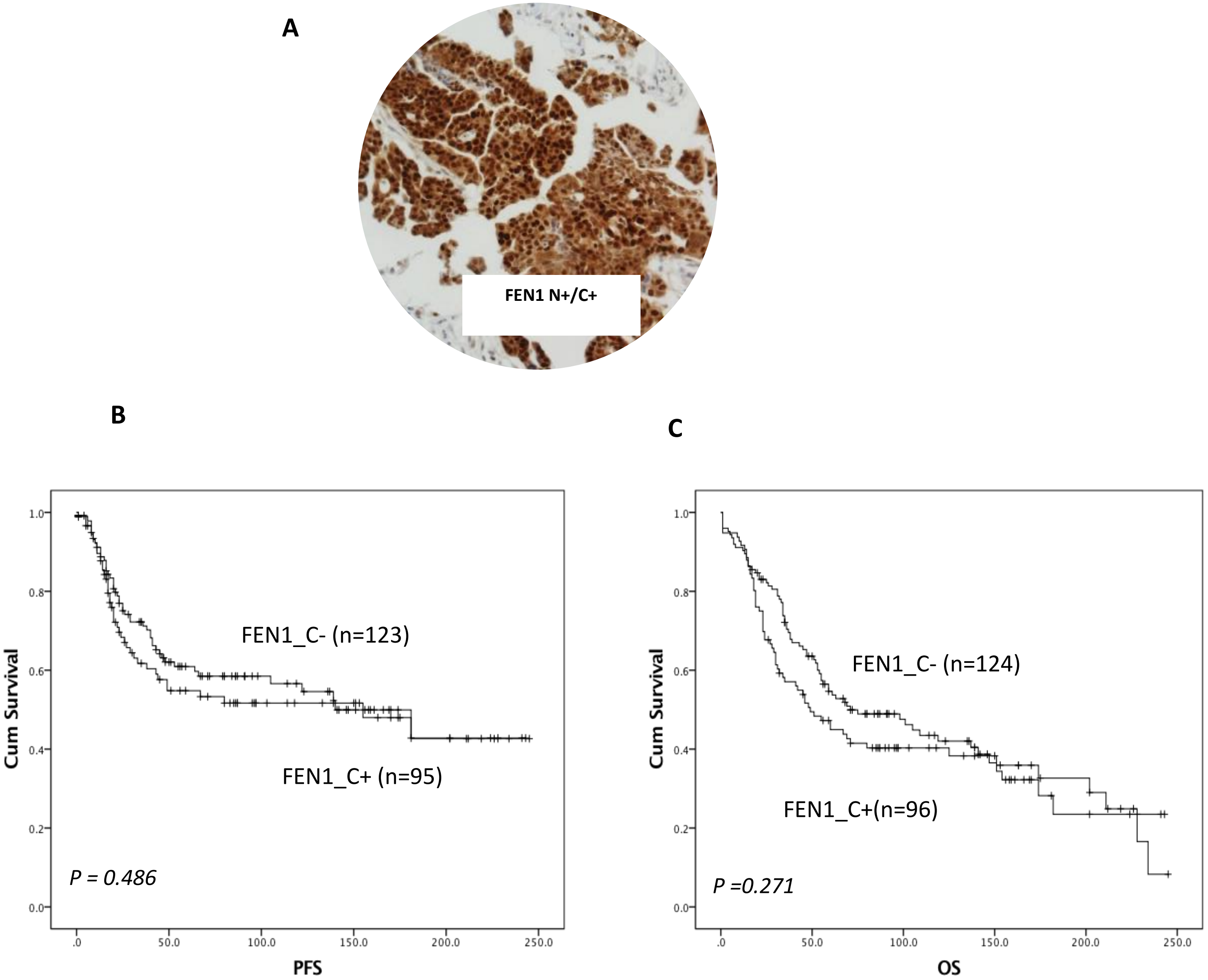

Supplementary Figure S2

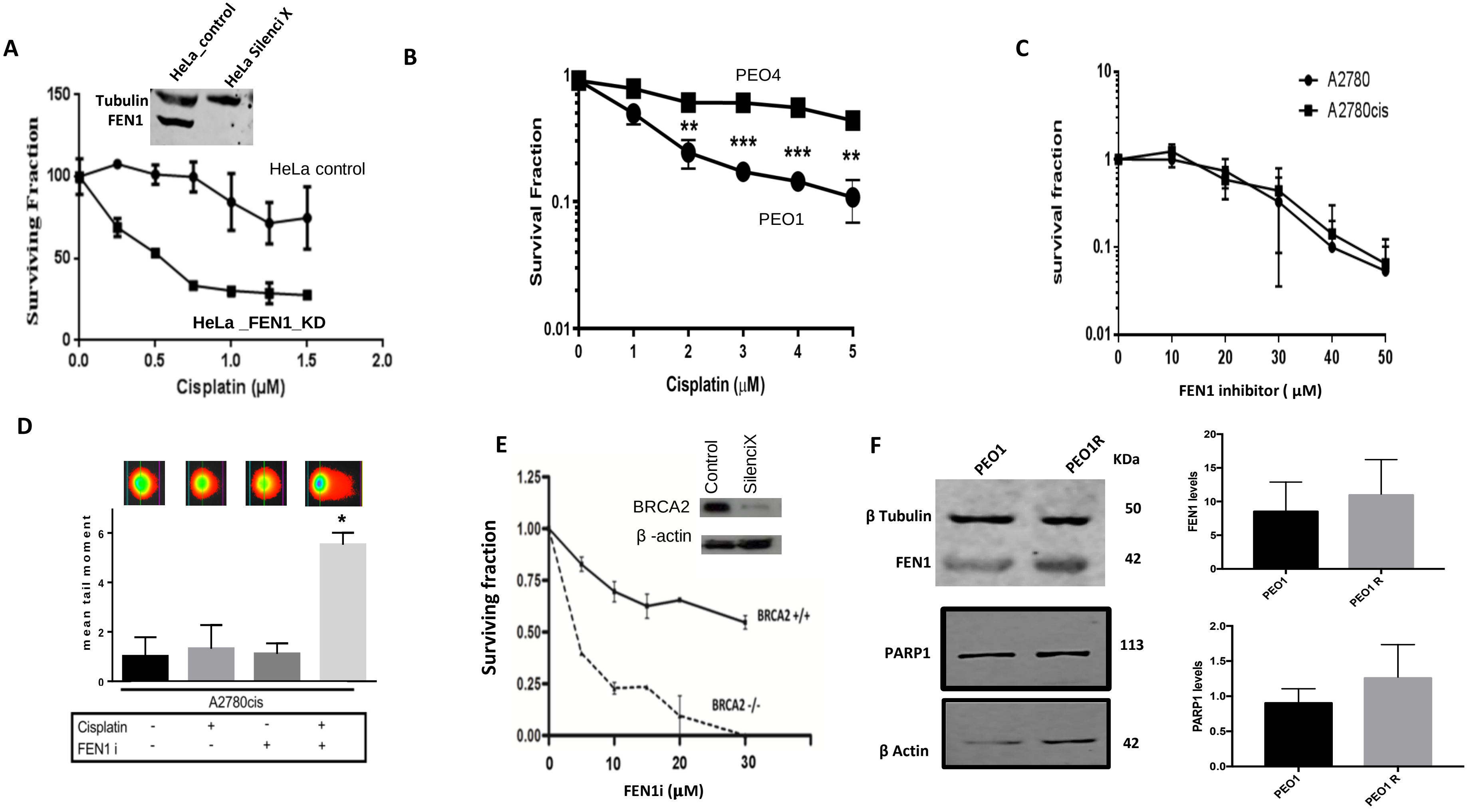

Supplementary Figure S3

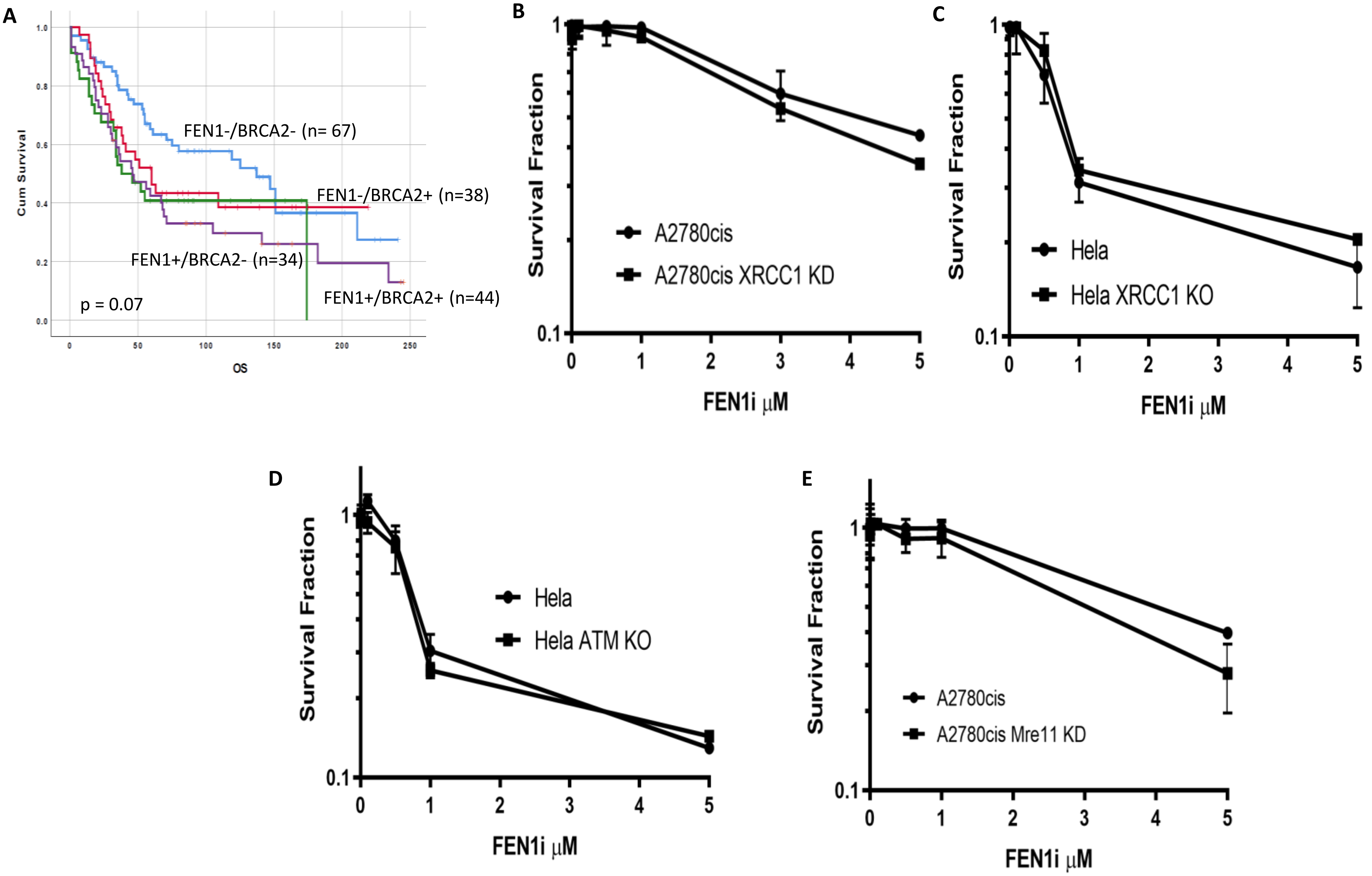

Supplementary Figure S4

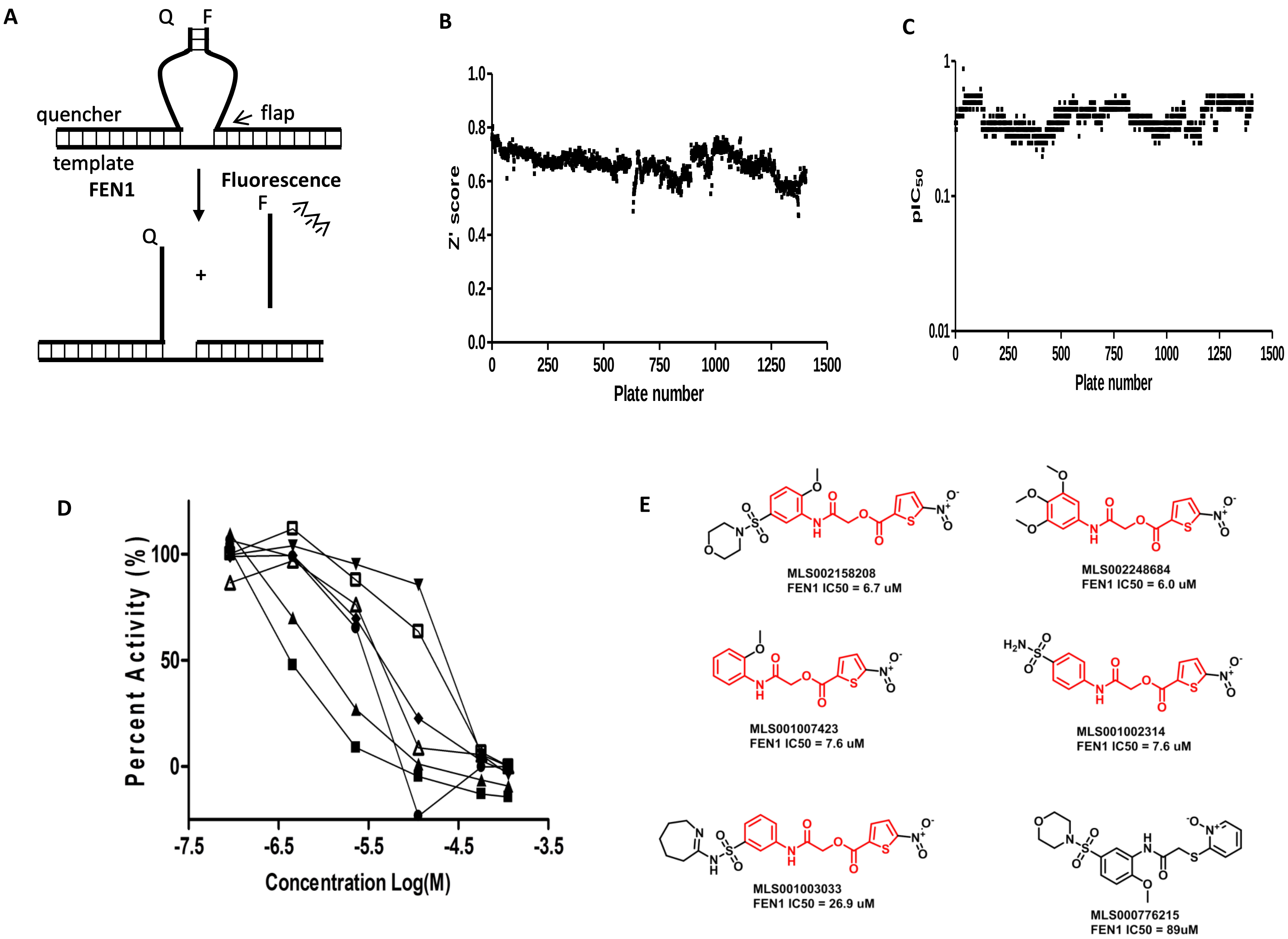

Supplement: Supplementary file 1 [file cancers-13-01866-s001.zip › cancers-1131740_supp.pdf]
