# Supplementary material for: FEN1 Blockade for Platinum Chemo-Sensitization and Synthetic Lethality in Epithelial Ovarian Cancers
Source: Cancers (Basel). 2021 Apr 14;13(8):1866. doi: 10.3390/cancers13081866 (PMC8070745; doi:10.3390/cancers13081866)

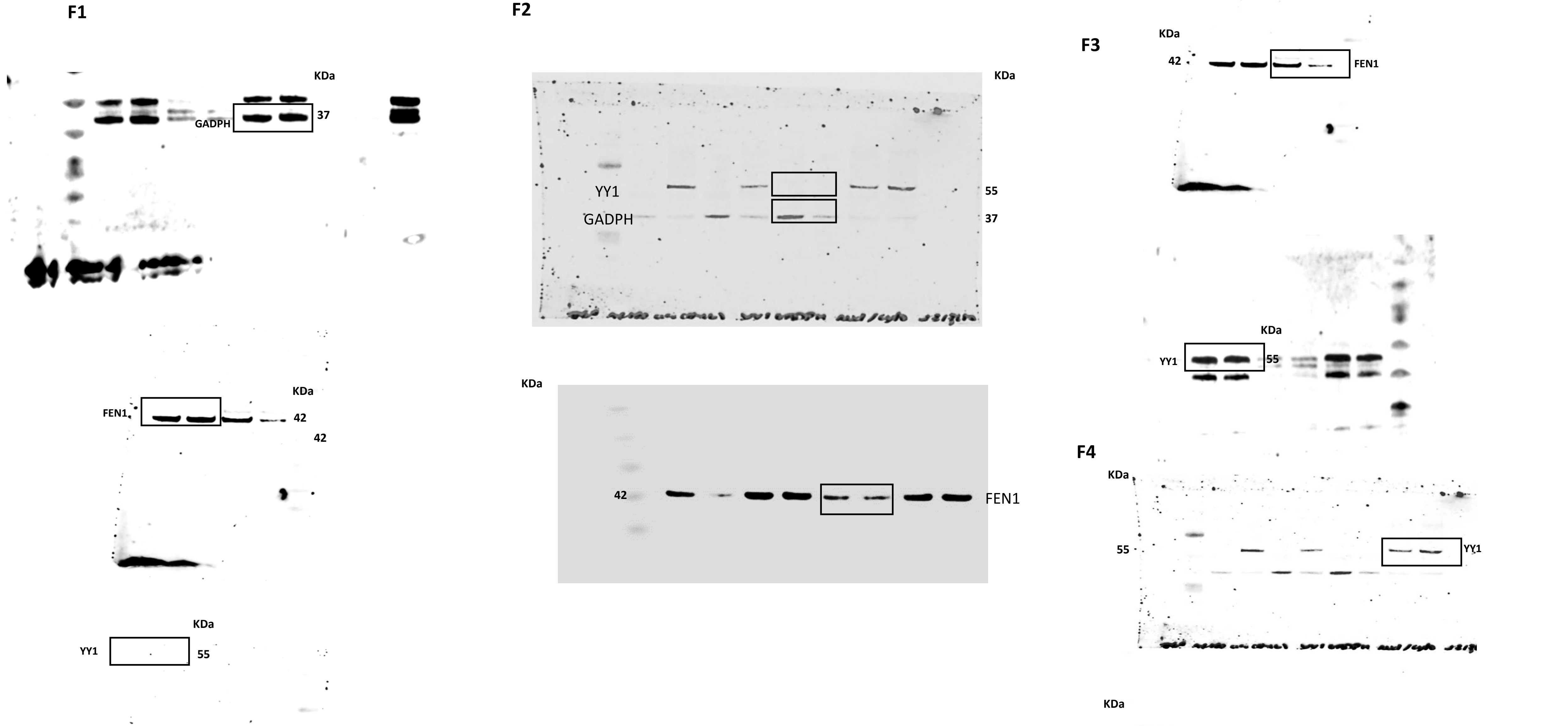
F1
KDa
GADPH
37
FEN1
KDa
42
42
KDa
YY1
55
F2
KDa
YY1
GADPH
55
37
KDa
42
FEN1
F3
KDa
42
FEN1
KDa
YY1
55
F4
KDa
55
YY1
KDa
42
FEN1

Figure 1

Figure 2A

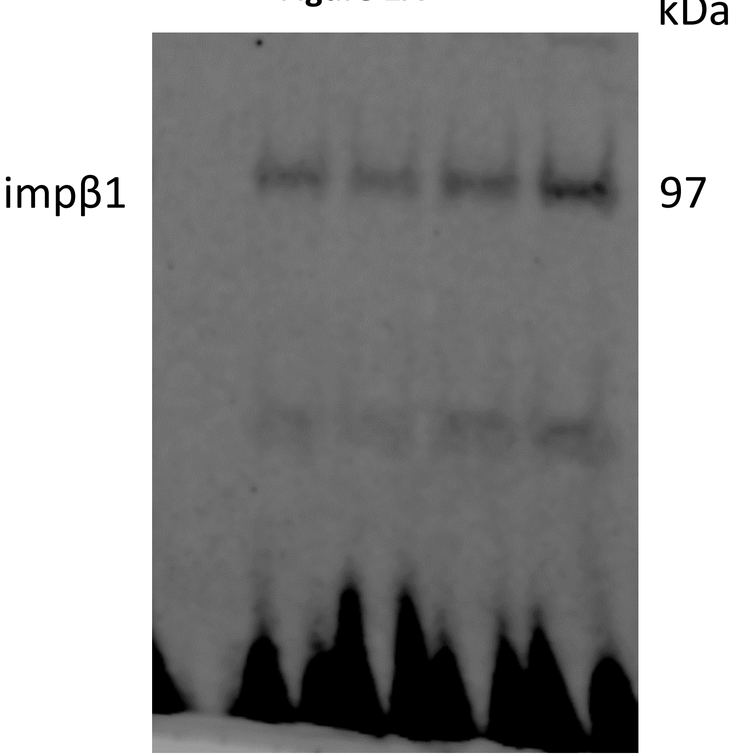

Figure 3A

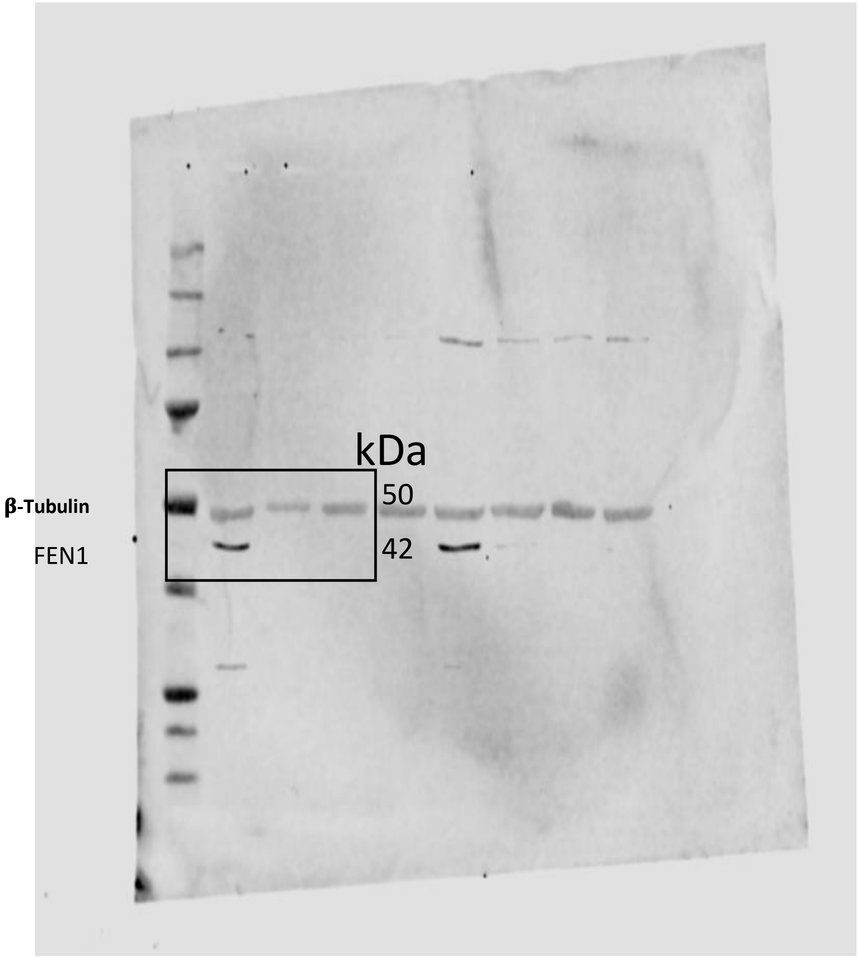

Figure 3F

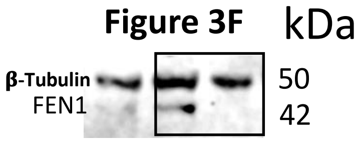

Figure 3F: repeat western blot

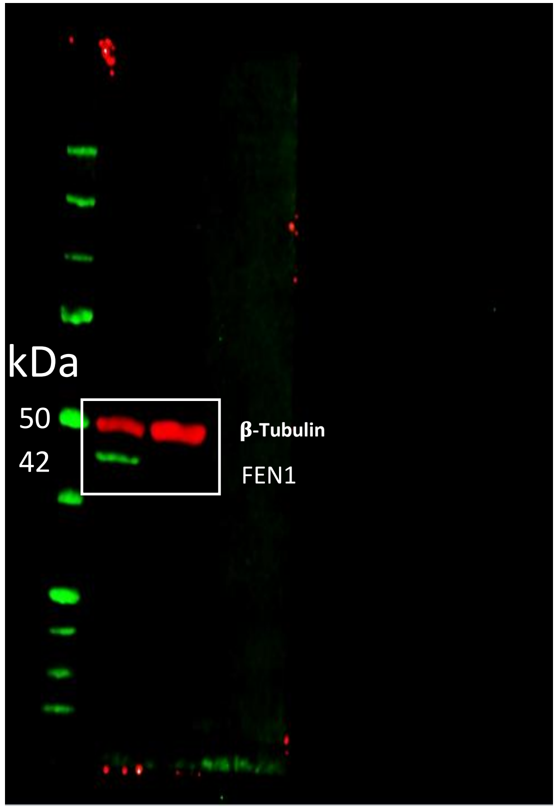

Figure 5A

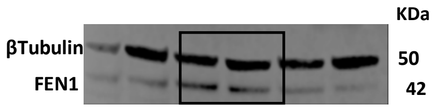

Figure 5B

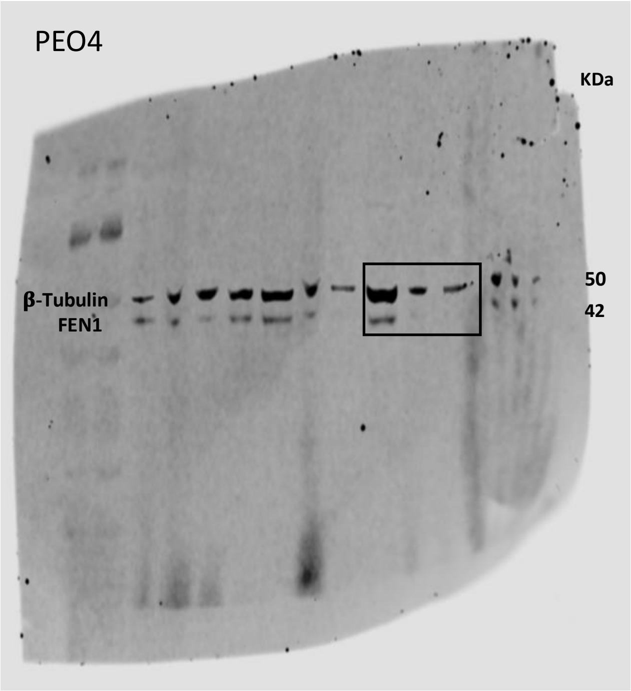

Figure 3M

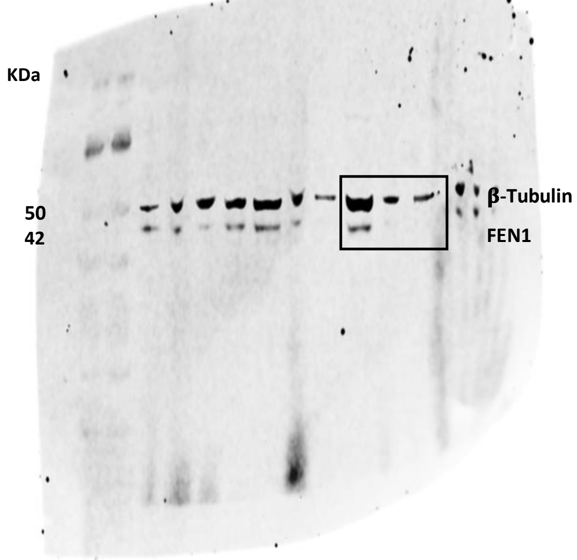

Figure 5B

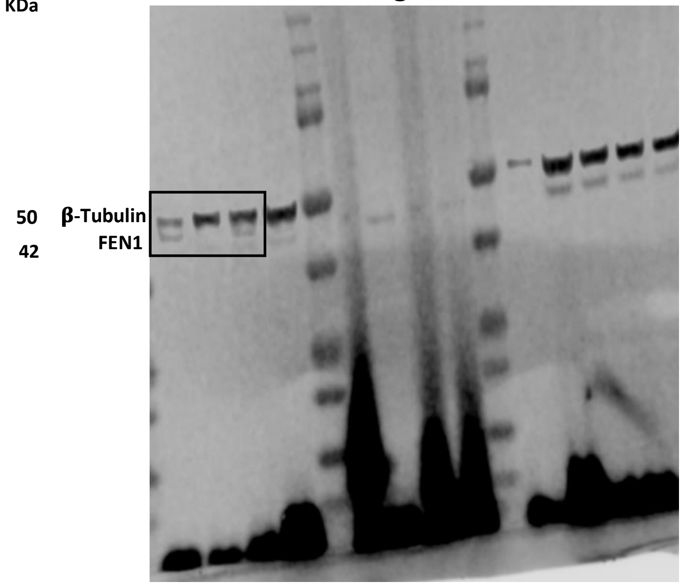

Figure 6K

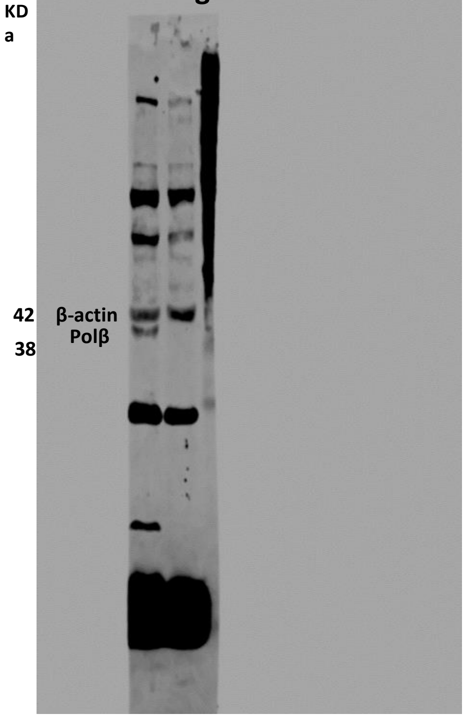

Figure 6H

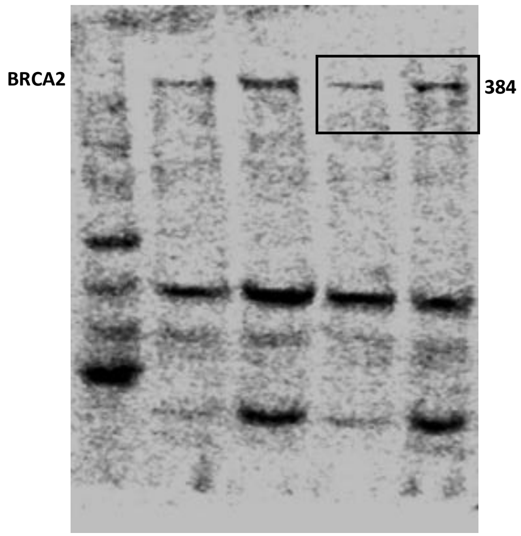

Figure 6I

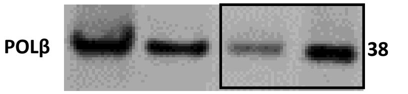

Figure 6J

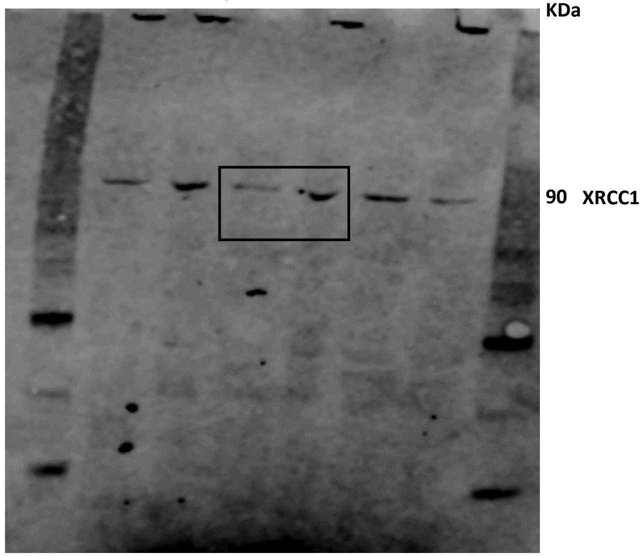

Figure 5A and 6H

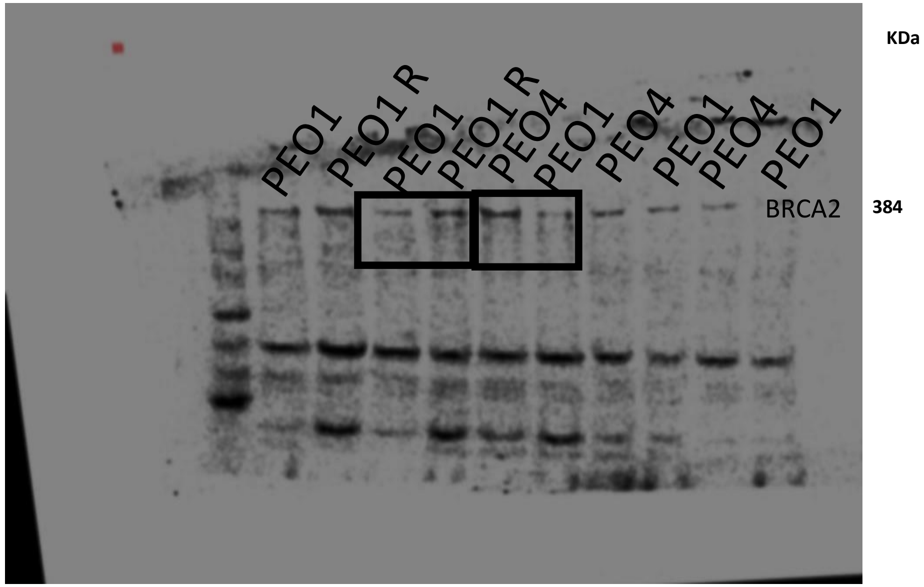

Figure 5A and 6H β actin only

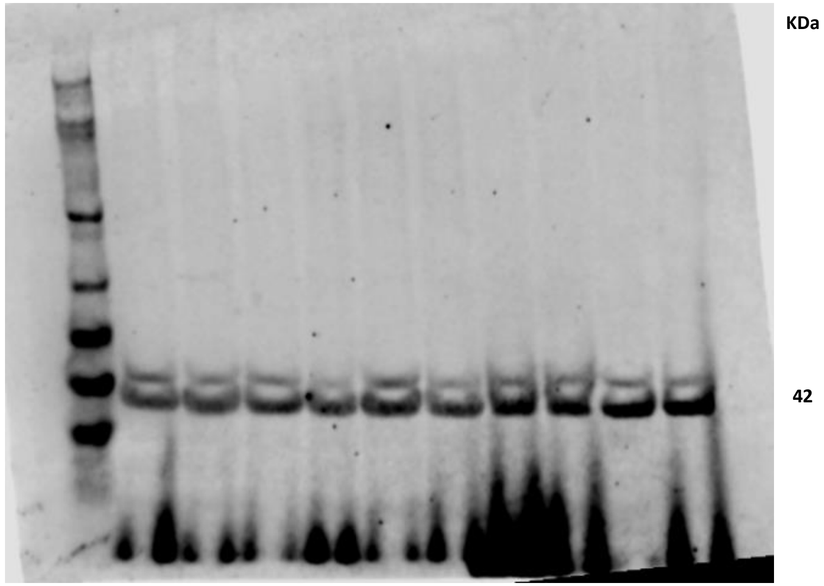

Figure 6I Polβ

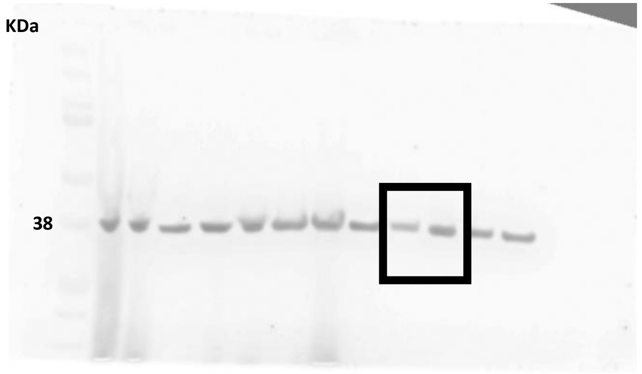

Figure 6I β Actin

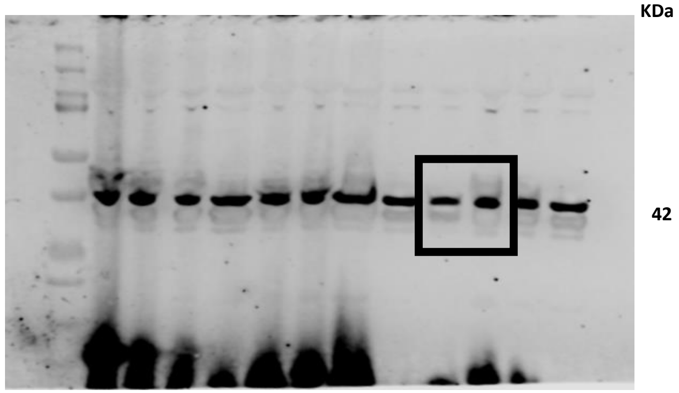

Figure 6J β Actin

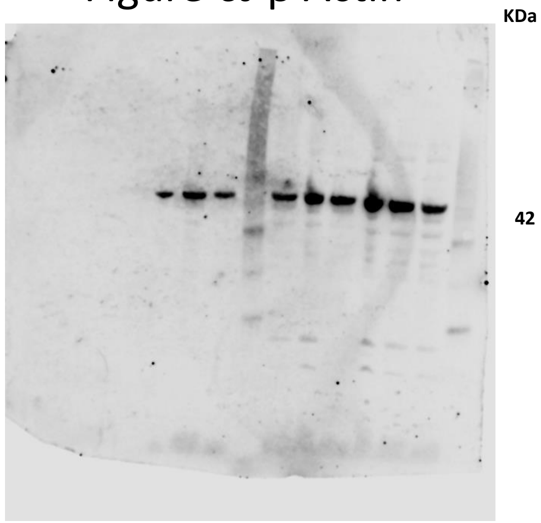

Figure S2A

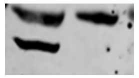

Figure S2A (another western blot)

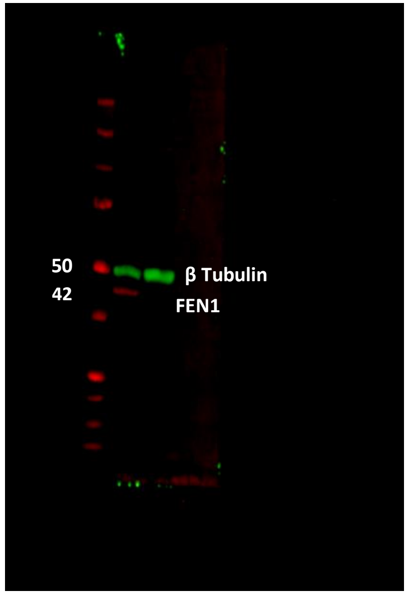

Figure S2E

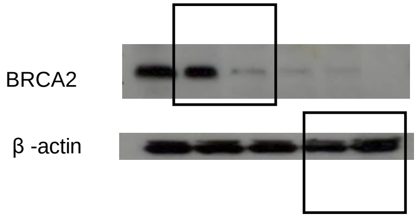

Figure S2E (another western blot)

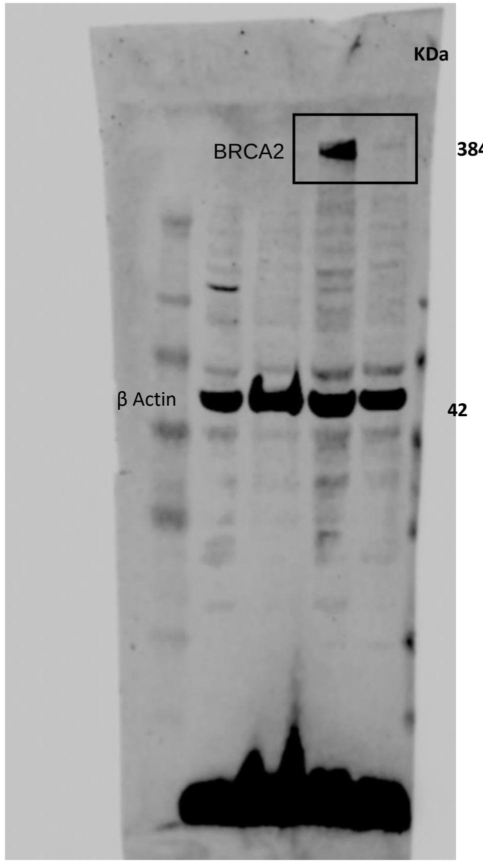

Figure S2F

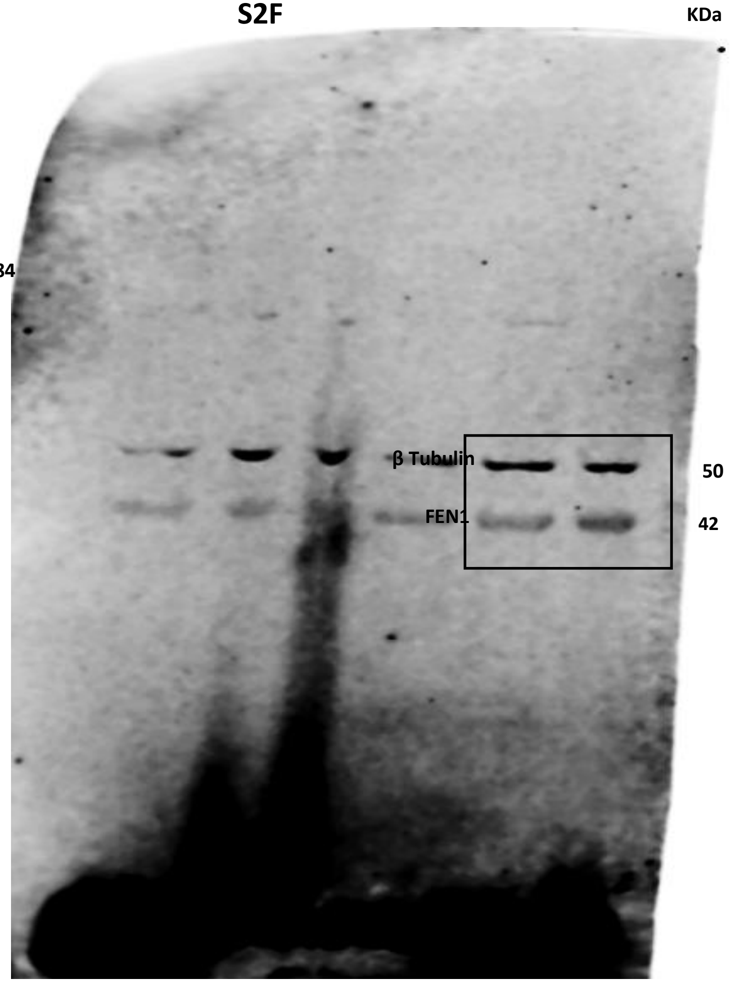

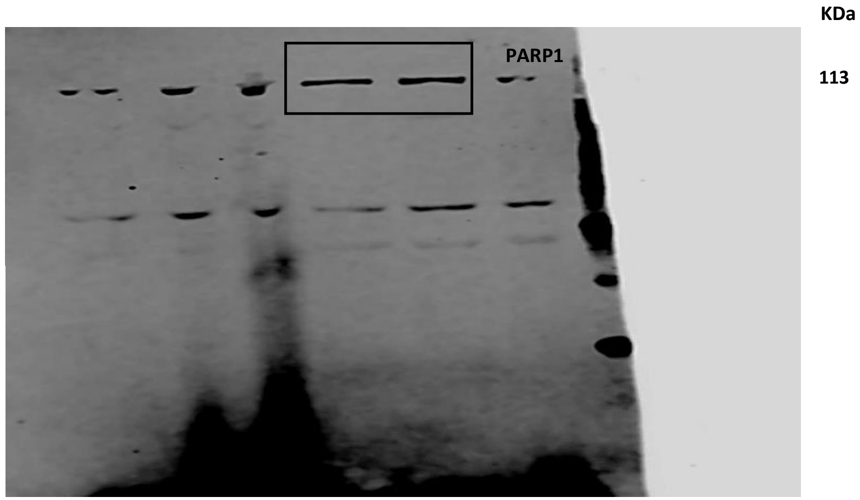

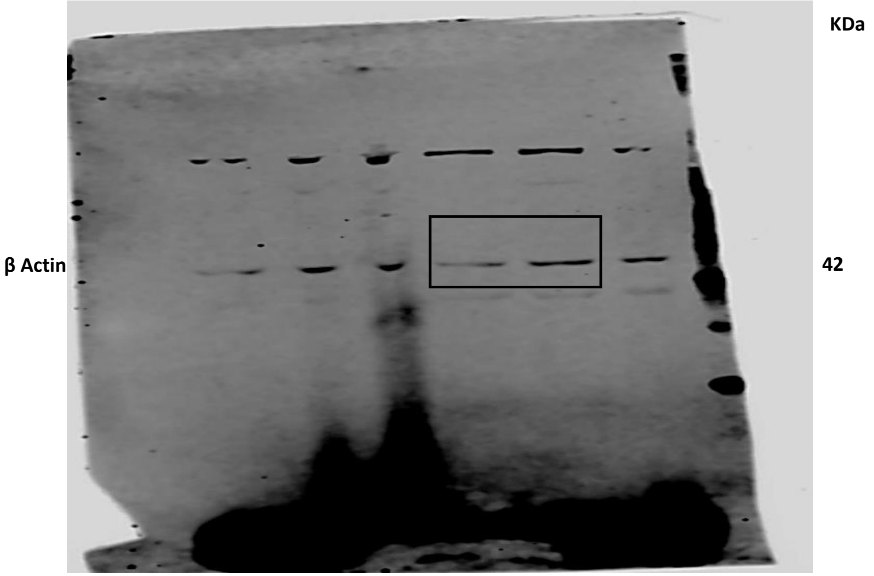

Supplement: Supplementary file 1 [file cancers-13-01866-s001.zip › Uncropped WB Figures.pdf]
